# Towards a Universal Measure of Complexity

**DOI:** 10.3390/e22080866

**Published:** 2020-08-06

**Authors:** Jarosław Klamut, Ryszard Kutner, Zbigniew R. Struzik

**Affiliations:** 1 Faculty of Physics, University of Warsaw, Pasteura 5, 02-093 Warsaw, Poland; jaroslaw.klamut@fuw.edu.pl (J.K.); z.r.struzik@p.u-tokyo.ac.jp (Z.R.S.); 2 Graduate School of Education, The University of Tokyo, 7-3-1 Hongo, Bunkyo-ku, Tokyo 113-0033, Japan; 3 Advanced Center for Computing and Communication, RIKEN, 2-1 Hirosawa, Wako, Saitama 351-0198, Japan

**Keywords:** dynamical complexity, universal complexity measure, irreversible processes, entropies, entropic susceptibilities

## Abstract

Recently, it has been argued that entropy can be a direct measure of complexity, where the smaller value of entropy indicates lower system complexity, while its larger value indicates higher system complexity. We dispute this view and propose a universal measure of complexity that is based on Gell-Mann’s view of complexity. Our universal measure of complexity is based on a non-linear transformation of time-dependent entropy, where the system state with the highest complexity is the most distant from all the states of the system of lesser or no complexity. We have shown that the most complex is the optimally mixed state consisting of pure states, i.e., of the most regular and most disordered which the space of states of a given system allows. A parsimonious paradigmatic example of the simplest system with a small and a large number of degrees of freedom is shown to support this methodology. Several important features of this universal measure are pointed out, especially its flexibility (i.e., its openness to extensions), suitability to the analysis of system critical behaviour, and suitability to study the dynamic complexity.

## 1. Introduction

Analysis of the concept of complexity is a non-trivial task due to its diversity, arbitrariness, uncertainty, and contextual nature [1,2,3,4,5,6,7,8,9,10]. There are many different levels/scales, faces, and types of complexity, researched with very different technologies/techniques and tools [11,12,13] (and refs. therein). In the context of dynamical systems, Grassberger suggested [14] that a slow convergence of the entropy to its extensive asymptotic limit is a signature of complexity. This idea was materialized [15,16] further by information and statistical mechanics techniques. It generalizes many previous approaches to complexity, unifying physical ideas with ideas from learning and coding theory [17]. There also exists a connection of this approach to algorithmic or Kolmogorov complexity. The hidden pattern can be the essence of complexity [18,19,20,21]. Techniques adapted from the theories of information and computation have led physical science (in particular, the region extended between classical determinism and deterministic chaos) to discover hidden patterns and quantify their dynamic structural complexity [22]. The above approaches are not universal—they only capture small fragments of the concept of complexity.

We must remember that complexity also depends on the conditions imposed (e.g., boundary or initial conditions), as well as the restrictions adopted. This creates a challenge for every complexity study. It concerns the complexity that can appear in the movement of a single entity and collection of entities braided together. These entities can be irreducible or straightforward, simple systems, but they can also be complex systems.

When we talk about complexity, we mean irreducible complexity, which can no longer be divided into smaller sub-complexities. We refer to this as a primary complexity. Considering the primary complexity here, we mean one that can be expressed at least in an algorithmic way—it is an effective complexity if it also contains a logical depth [23,24,25,26,27]. We should take into account that our models (analytical and numerical) and theories describing reality are not fully deterministic. The evolution of a complex system is potentially multi-branched and the selection of an alternative trajectory (or branch selection) is based on decisions taken randomly.

One of the essential questions concerning a complex system is the problem of its stability/robustness and the question of the stationarity of its evolution [28]. Moreover, the relationship between complexity and disorder on the one hand, and complexity and pattern on the other is an important question—especially in the context of irreversible processes, where non-linear processes, running away from the equilibrium, play a central role. Financial markets can be a spectacular example of these processes [29,30,31,32,33,34,35,36,37,38,39].

The central question of whether entropy is a direct measure of complexity is one we answer in the negative. In our opinion, based on the Gell–Mann concept of complexity, the measure of complexity is appropriately, non-linearly transformed entropy. This work is devoted to finding this transformation and examining the resulting consequences.

## 2. Definition of a Universal Measure of Complexity and Its Properties

In this Section, we translate the Gell–Mann general qualitative concept of complexity into the language of mathematics, and we present the consequences of this.

### 2.1. The Gell–Mann Concept of Complexity

The problem of defining a universal measure of complexity is urgent. For this work, the Gell–Mann concept [23,40] of complexity is the inspirational starting point. We apply this concept to irreversible processes, by assuming that both fully ordered and fully disordered systems cannot be the complex. The fully ordered system essentially has no complexity because of maximal possible symmetry of the system, but the fully disordered system contains no information as it entirely dissipates. Hence, the maximum of complexity should be sought somewhere in between these pure extreme states. This point of view allows for the introduction of a formal quantitative phenomenological complexity measure based on entropy as a parameter of order [29,41]. This measure reflects the dynamics of the system through the dependence of entropy on time. The vast majority of works analyzing the general aspects of complexity, including its basis, are based on information theory and computational analysis. Such an approach requires supplementing with a provision allowing a return from a bit representation to physical representation—only this will allow physical interpretations, including understanding of the causes of complexity.

We define the phenomenological partial measure of complexity as a non-linear function of entropy *S* of the order of (m,n),

(1) $$\begin{array}{ccc} CX(S;m,n) & \overset{\mathrm{def}.}{=} & {\left(S^{max}-S\right)}^{m}{\left(S-S^{min}\right)}^{n}\\ & = & CX\left(S;m-1,n-1\right)\left[{\left(\frac{Z}{2}\right)}^{2}-{\left(S-S^{arit}\right)}^{2}\right],\;m,n\geq 1, \end{array}$$

where $S^{min}$ and $S^{max}$ are minimal and maximal values of entropy *S*, respectively, $S^{arit}=\frac{S^{min}+S^{max}}{2}$, and the entropic span $Z\overset{\mathrm{def}.}{=}S^{max}-S^{min}$, whereas *m* and *n* are natural numbers (an extension to real positive numbers is possible but this is not the subject of this work). They define the order (m,n) of the partial measure of complexity CX. Let us add that this formula is also applicable at a mesoscopic scale. In other words, complexity appears in all systems for which we can build entropy. Notably, $S^{max}$ does not have to concern the state of thermodynamic equilibrium of the system. It may refer to the state for which entropy reaches its maximum value in the observed time interval. However, in this work, we are only limited to systems having a state of thermodynamic equilibrium. Below, we discuss the Equation (1), indicating that it satisfies all properties of the measure of complexity. Of course, when m=0 and n=1 then CX simply becomes $S-S^{min}$, i.e., the entropy of the system (the constant is not important here). However, when m=1,n=0, we obtain the information contained in the system (constant does not play a role here). Equation (1) gives us a lot more—showing this is the purpose of this work (helpful features of *CX* are shown in Appendix A).

The partial measure of complexity given by Equation (1) is determined with the accuracy of the additive constant of *S*, i.e., this constant does not contribute to the measure of the complexity of the system.

Using Equation (1), we can also enter the partial measure of specific complexity, as follows,

(2) $$\begin{array}{c} cx\left(s;m,n\right)\overset{\mathrm{def}.}{=}\frac{1}{N^{m+n}}CX\left(Ns;m,n\right), \end{array}$$

where *N* is the number of entities that make up the system and specific entropy s=S/N. As one can see, the partial measure of specific complexity cx is independent of *N* for an extensive system. Specific entropy and specific complexity are particularly convenient when comparing different extensive systems and when we do not examine the complexity dependence on *N*.

However, the extraction of an additional multiplicative constant (e.g., particle number) to have *s* independent of *N* often presents a technical difficulty, or may even be impossible, especially for non-extensive systems. Then it is more convenient to use the entropy of the system instead of the specific entropy. It is also important to realize that determining extreme entropy values (or extreme specific entropy values) of actual systems can be complicated and it requires additional dedicated tools/technologies, algorithms, and models.

The partial measures of complexity are enslaved by entropy in every order (m,n) of complexity. However, the kind of entropy we use in Equation (1) depends on the specific situation of the system and what we want to know about the system, because our definition of complexity does not specify this. From our point of view, relative entropies formulated in the spirit of Kullback–Leibler seem to be the most appropriate (this is referred to in Appendix B). Using the Kullback–Leibler type of entropy, one can express both ordinary entropies and conditional entropies, in particular one can describe the entropy rate increasingly used in the context of complexity analysis.

The entropy here can be both additive (the Boltzmann–Gibbs thermodynamic one [42], Shanon information [17], Rényi [43]), and non-additive entropy (Tsallis [44]). The measure CX(S) is a concave (or convex up) function of entropy *S*, which disappears on the edges at points $S=S^{min}$ and $S=S^{max}$.

It has a maximum

(3) $$\begin{array}{c} CX^{max}=CX\left(S=S_{CX}^{max};m,n\right)=m^{m}\;n^{n}{\left(\frac{Z}{m+n}\right)}^{m+n} \end{array}$$

at point

(4) $$\begin{array}{c} S=S_{CX}^{max}=\bar{S}=\frac{mS^{min}+nS^{max}}{m+n}=\frac{\frac{1}{m}S^{max}+\frac{1}{n}S^{min}}{\frac{1}{m}+\frac{1}{n}} \end{array}$$

as at this point $\frac{dCX(S)}{dS}{|}_{S=\bar{S}}\;=\;0$ and $\frac{d^{2}CX\left(S\right)}{dS^{2}}{|}_{S=\bar{S}}\;<\;0$. The quantity $S_{CX}^{max}$ is a characteristic also because it is a weighted average. The quantity $CX^{max}$ is well suited to global universal measurements of complexity, because (at a given order (m,n)), it only depends on the entropy span *Z*. The quantity $cx^{max}\overset{\mathrm{def}.}{=}CX^{max}/N^{m+n}$ might also be a good candidate for measuring the logic depth of complexity.

### 2.2. The Most Complex Structure

The question now arises about the structure of the system corresponding to entropy $S_{CX}^{max}$ given by Equation (4). The answer is given by the following constitutive equation,

(5) $$\begin{array}{c} S\left(Y=Y^{CX^{max}}\right)=S_{CX}^{max}, \end{array}$$

where *Y* is the set of variables and parameters (e.g., thermodynamic), on which the state of the system depends. However, $Y=Y^{CX^{max}}$ is a set of such values of these variables and parameters that are the solution of Equation (5). This solution gives the entropy value $S=S_{CX}^{max}$ that maximizes the partial measure of complexity, that is $CX=CX^{max}$. Hence, with the value of $Y^{CX^{max}}$, we can finally answer the key question: what structure/pattern is behind $CX^{max}$ or what the structure of maximum complexity looks like.

There are a few comments to be made regarding the constitutive Equation (5) itself. It is a (non-linear) transcendental equation in the untangled form relative to the *Y*. This equation should be numerically solved, because we do not expect it to have an analytical solution for maximally complex systems. An instructive example of a specific form of this equation and its solution for a specific physical problem is presented in Section 3. However, this will help us to understand how our machinery works.

Equation (4) legitimizes the measure of complexity we have introduced. Namely, its maximum value falls on the weighted average entropy value, which describes the optimal mixture of completely ordered and completely disordered phases. To the left of $\bar{S}$, we have a phase with dominance of order and to the right a phase with dominance of disorder. The transition between both phases at $\bar{S}$ is continuous. Thus, we can say that the partial measure of complexity that we have introduced also defines a certain type of phase diagram in *S* and CX variables (phase diagram plain). Section 2.5 provides more detailed information.

### 2.3. Evolution of the Partial Measure of Complexity

Differentiating Equation (1) over time *t*, we obtain the following non-linear dynamics equation,

(6) $$\begin{array}{c} \frac{dCX(S(t);m,n)}{dt}=\chi_{CX}\left(S;m,n\right)\;\frac{dS(t)}{dt}=\left(m+n\right)\left[S_{CX}^{max}-S\left(t\right)\right]CX\left(S\left(t\right);m-1,n-1\right)\frac{dS(t)}{dt}, \end{array}$$

where the entropic *S*-dependent (non-linear), susceptibility is defined by

(7) $$\begin{array}{c} \chi_{CX}\left(S;m,n\right)\overset{\mathrm{def}.}{=}\frac{\partial CX(S;m,n)}{\partial S}=\left(m+n\right)\left[S_{CX}^{max}-S\left(t\right)\right]CX\left(S\left(t\right);m-1,n-1\right) \end{array}$$

and $\frac{dS(t)}{dt}$ can be expressed, for example, using the right-hand side of the master Markov equation (see Ref. [45] for details). However, we must realize that the dependence of entropy on time can, in general, be non-monotonic, because real systems are not isolated (cf. the schematic plot in Figure 2). One can see how the dynamics of complexity is controlled in a non-linear way by the evolution of the entropy of the system.

In concluding this Section, we state that Equations (1)–(6) together provide a technology for studying the multi-scale aspects of complexity, including the dynamic complexity. However, it is still a simplified approach, as we show in Section 4.

### 2.4. Significant Partial Measure of Complexity

**Figure 1:**
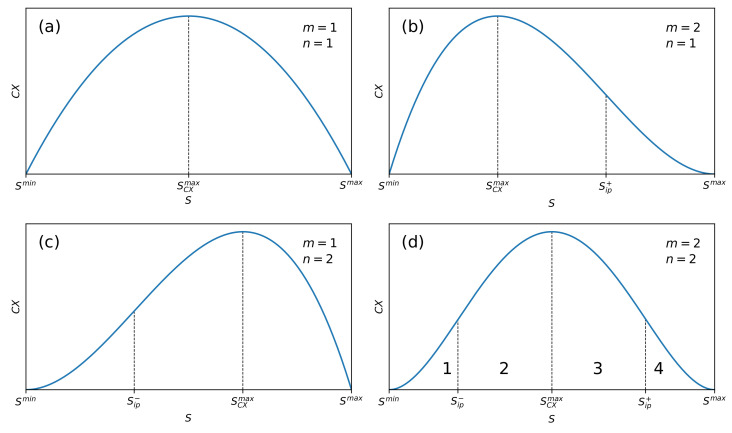
Plots of the partial measure of complexity CX(S;m,n) vs. *S* given by Equation (1) for four characteristic cases: (**a**) Case n=m=1 where no inflection points, $S_{ip}^{\mp }$ are present. (**b**) Case m=2 and n=1 where a single inflection point $S_{ip}^{+}$ is present. (**c**) Case m=1 and n=2 where a single inflection point $S_{ip}^{-}$ is present. (**d**) Case m=2 and n=2 where both inflection points are present. The shape of the curve, containing two inflection points, is typical for partial measures of complexity, characterized by exponents m,n≥2. Numbers 1–4 mark individual phases differing in the degree of order.

We consider the partial measure of complexity to be significant when the entropy of the system is located between two inflection points of the CX(S;m,n) curve, i.e., in the range $S_{ip}^{-}\leq S\leq S_{ip}^{+}$. This case occurs for n,m≥2. We then obtain

(8) $$\begin{array}{c} S^{min}<S_{ip}^{\mp }=S^{min}+\frac{n(n-1)}{\sqrt{n(n+m-1)}}\frac{S^{max}-S^{min}}{\sqrt{n(n+m-1)}\pm \sqrt{m}}<S^{max}, \end{array}$$

see Figure 1d for details.

There are two different cases where a single inflection point is present. Namely,

(9) $$\begin{array}{cc} S^{min}<S_{ip}^{-}=\frac{2S^{max}+m\left(m-1\right)S^{min}}{2+m(m-1)}<\bar{S}, & \mathrm{for}\;m\geq 2,\;n=1, \end{array}$$

and

(10) $$\begin{array}{cc} \bar{S}<S_{ip}^{+}=\frac{2S^{min}+n\left(n-1\right)S^{max}}{2+n(n-1)}<S^{max}, & \mathrm{for}\;m=1,\;n\geq 2. \end{array}$$

In Figure 1b, we present the case defined by Equation (9), while that defined by Equation (10) is shown in Figure 1c.

For n=m=1, the curve CX(S;m,n) vs. *S* has no inflection points and it looks like a horseshoe (cf. Figure 1a).

Notably, we can equivalently write

(11) $$\begin{array}{cc} S^{min}<S_{ip}^{\mp }=S^{max}-\frac{m(m-1)}{\sqrt{m(n+m-1)}}\frac{S^{max}-S^{min}}{\sqrt{m(n+m-1)}\mp \sqrt{n}}<S^{max}, & \mathrm{for}\;n,m\geq 2. \end{array}$$

Let us consider the span $Z_{ip}=S_{ip}^{+}-S_{ip}^{-}$ of the two-phase area. From Equation (8), or equivalently from Equation (11), we obtain

(12) $$\begin{array}{c} Z_{ip}=\frac{2\sqrt{nm}}{\left(n+m\right)\sqrt{n+m-1}}Z. \end{array}$$

As one can see, the span $Z_{ip}$ depends linearly on the span *Z* and in a non-trivial way on the exponents *n* and *m*. Thus, with the *Z* set, only $Z_{ip}$’s non-trivial dependence on the order (m,n) of measure of complexity CX occurs, which is different from $CX^{max}$ dependence. In other words, $Z_{ip}$ is less sensitive to complexity than $CX^{max}$.

The significant partial measure of complexity ranges between the two inflection points only for the case n,m≥2 (cf. Figure 1d). Indeed, a mixture of phases is observed in this area. For areas where $S^{min}\leq S<S_{ip}^{-}$ and $S_{ip}^{+}<S\leq S^{max}$, we have (practically speaking) only single phases, ordered and disordered, respectively (see Section 2.5 for details). The case defined by Equation (8), and equivalently by Equation (11), is the most general, while taking into account the fullness of complexity behaviour as a function of entropy. Other cases impoverish the description of complexity. Therefore, we will continue to consider the situation, where n,m≥2.

The choice of any of the CX(S;m,n) forms (i.e., exponents *n* and *m*) is a somewhat arbitrary function of the state of the system as it depends on the function of the state, that is on the entropy. In our opinion, the shape of the CX(S;m,n) measure vs. *S* we present in Figure 1d is the most appropriate, because only then the significant complexity is ranging between non-vanishing inflection points $S_{ip}^{-}$ and $S_{ip}^{+}$.

In generic case we should, however, use the series of partial measures defined by Equation (1). Then, we define the order of the partial complexity using the pair of exponents (n,m). The introduction of the order of the partial complexity is in line with our perception of the existence of multiple levels of (full) complexity.

**Figure 2:**
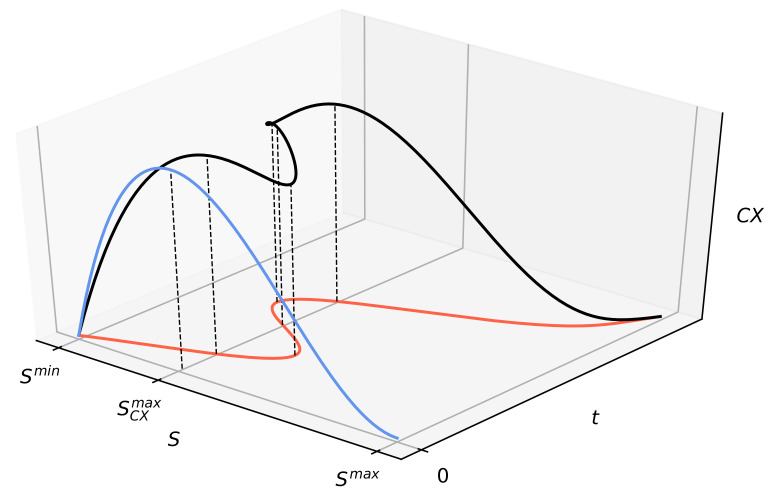
Schematic plot of the partial measure of complexity CX(S;m,n) vs. *S* and *t* given by Equation (1). The red curve shows the dependence of entropy *S* on time *t*. The black curve represents CX(S(t);m,n) in three dimensions. The blue curve represents projection of the black curve on the (S,CX) plane. We show different variants of this blue curve presented in Figure 1. The non-monotonic dependence of the entropy on time visible here indicates the open nature of the system. However, this non-monotonicity is not visible through the blue curve. For instance, the three local maxima of the black curve collapse to one of the blue curve.

We are able to discover the nature of the CX measure, i.e., its dynamics and, in particular, its dynamical structures, when we analyze the entropy dynamics S(t) (see Figure 2 for details).

The measurability of the partial measure of complexity is necessary for characterizing it quantitatively and to be able to compare different complexities. Following Gell–Mann [40], we must identify the scales at which we perform the analysis and thus determine coarse-graining to define the entropy. Its dependence on complexity cannot be ruled out.

However, the question of direct measurement of the partial measure of complexity in an experiment (real or numerical) remains a challenge.

### 2.5. Remarks on the Partial Entropic Susceptibility

An essential tool for studying phase transitions is the system susceptibility—in our case, the partial entropic susceptibility of the partial measure of complexity. Here, it (additionally) plays the role of the partial order parameter.

**Figure 3:**
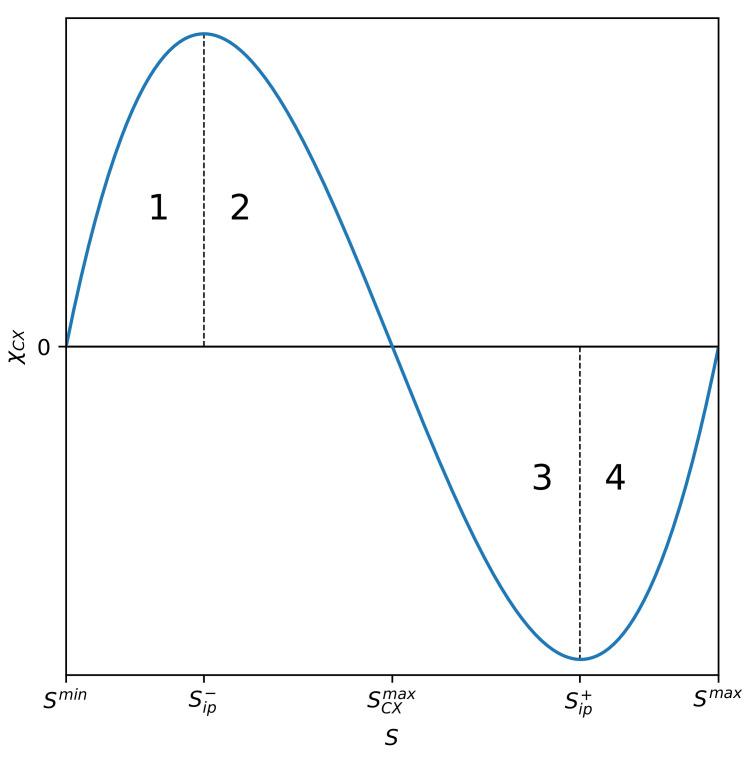
Plot of the partial entropic (non-equilibrium) susceptibility $\chi_{CX}\left(S;m,n\right)$ of the partial measure of complexity vs. *S* given by Equation (7) at fixed order (m=2,n=2). The finite susceptibility value at the $S_{ip}^{-}$ and $S_{ip}^{+}$ phase transition points (cf. Figure 1) may be considered to correspond to finite susceptibility value in absorbing the non-equilibrium phase transition in the model of direct percolation at a critical point in the presence of an external field [21]. However, the situation presented here is richer, because susceptibility changes its sign, smoothly passing through zero at $S=S_{CX}^{max}$. At this point, the system is exceptionally robust and, therefore, is poorly affected by data artefacts, because its susceptibility vanishes there.

The plot of susceptibility $\chi_{CX}\left(S;m,n\right)$ vs. *S* is presented in Figure 3. Four phases, already marked in Figure 1, are visible (also numbered 1 to 4).

**Figure 4:**
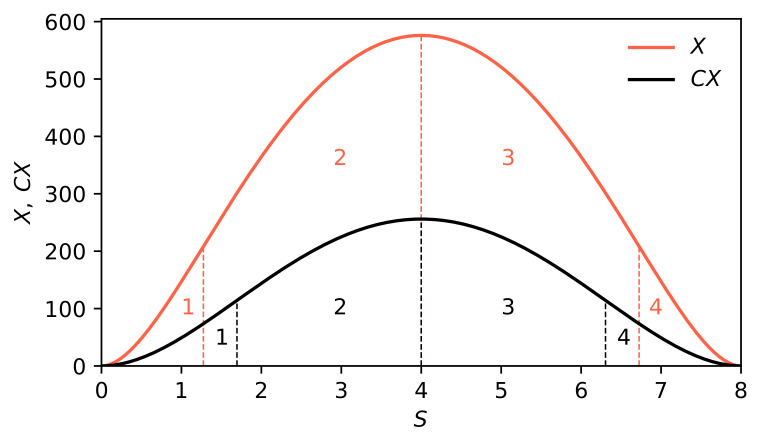
Comparison of the partial measure of complexity CX(S;m=2,n=2) given by Equation (1) and full measure of complexity $X(S;m_{0}=2,n_{0}=2)$ given by Equation (17), for instance, for the symmetric case of $m=n=m_{0}=n_{0}$. In addition, we assume that $S^{min}=0,S^{max}=8$ and M=10. Vertical dashed lines indicate inflection points: black for the CX curve, orange for the *X* curve, while $S_{CX}^{max}=S_{X}^{max}=4$. Notably, $S_{X}^{max}$ maximizes *X* (here at a given ratio Z/M=0.8). Vertical dashed lines mark the locations of inflection points on both curves.

**Figure 5:**
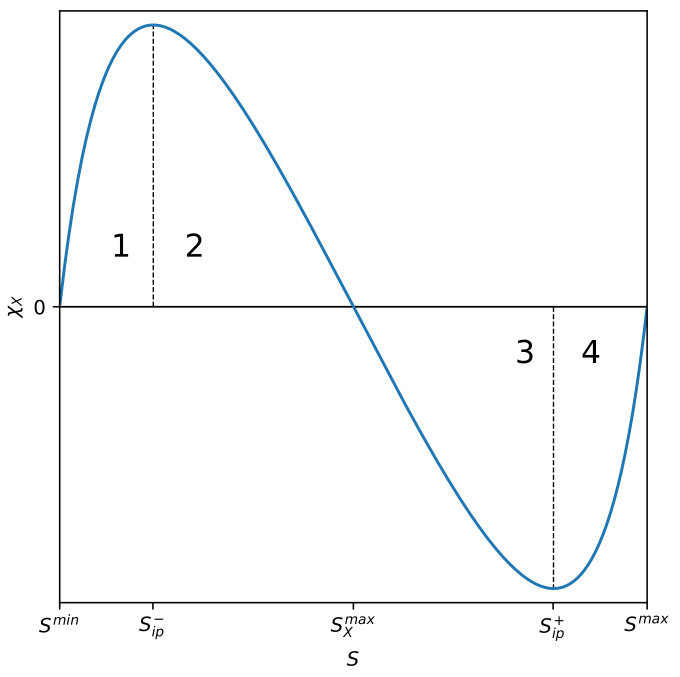
Plot of the full entropic susceptibility $\chi_{X}\left(S;m_{0},n_{0}\right)$ of the full measure of complexity vs. *S* given by Equation (19), at arbitrary fixed order $\left(m_{0}=2,n_{0}=2\right)$. As expected from the comparison with Figure 3, the turning points of CX (cf. Figure 4) lie within the *S* interval bounded by inflection points of *X*.

Phase number 1 is almost entirely ordered—the disordered phase input is residual. At point $S_{ip}^{-}$, there is a phase transition to the mixed-phase marked with number 2, still with the predominance of the ordered phase. At the $S_{ip}^{-}$ inflection point, the entropic susceptibility reaches a local maximum. By further increasing the entropy of the system, it enters phase 3 as a result of phase transition at the very specific $S_{CX}^{max}$ transition point. At this point, the entropic susceptibility of the partial measure of complexity disappears. This mixed phase (number 3) is already characterized by the advantage of the disordered phase over the ordered one. Finally, the last transition, which occurs at $S_{ip}^{+}$, leads the system to the dominating phase of the disordered phase—the input of the ordered phase is residual here. At this transition point, the susceptibility reaches a local minimum. Intriguingly, entropic susceptibility can have both positive and negative value passing smoothly through zero at $S=S_{CX}^{max}$, where the system is exceptionally robust. The presence of phases with positive and negative entropic susceptibility is an exceptionally intriguing phenomenon. The phases discussed above, together with the above-mentioned inflection points, are also marked in Figure 1d. Let us add that the location of the phases mentioned above, i.e., the location of the inflection points, depends on the order (m,n) of the partial measure of complexity. This is clearly seen in Figure 4 and Figure 5.

The values of local extremes of the entropic susceptibility of the partial measure of complexity are finite here and not divergent, as in the case of (equilibrium and non-equilibrium) phase transitions in the absence of an external field. We use this definition to describe the critical behaviour of a system that we demonstrate in Section 2.7, where it requires an explicit dependence on *N*.

### 2.6. Universal Full Measure of Complexity

The full universal measure of complexity *X* is a weighted sum of the partial measures of complexity CX(S;m,n) for individual scales. That is,

(13) $$\begin{array}{c} X\left(S;m_{0},n_{0}\right)=\sum_{m\geq m_{0},n\geq n_{0}}w\left(m,n\right)CX\left(S;m,n\right),\;m_{0},n_{0}\geq 0, \end{array}$$

where w(m,n) is a normalized weight, which must be given in an explicit form. This form is to some extent imposed by the power-law form of partial complexity. Namely, we can assume

(14) $$\begin{array}{c} w\left(m,n\right)={\left(1-\frac{1}{M}\right)}^{2}\frac{1}{M^{m-m_{0}+n-n_{0}}},\;M>1, \end{array}$$

which seems to be particularly simple because

(15) $$\begin{array}{c} \frac{w(m+1,n)}{w(m,n)}=\frac{w(m,n+1)}{w(m,n)}=\frac{1}{M}, \end{array}$$

independently of *m* and *n*.

As one can see, Equation (13), supported by Equation (15), is the product of the sums of two geometric series,

(16) $$\begin{array}{ccc} X(S;m_{0},n_{0}) & = & {\left(S^{max}-S\right)}^{m_{0}}\left(1-\frac{1}{M}\right)\sum_{m\geq m_{0}}\frac{{\left(S^{max}-S\right)}^{m-m_{0}}}{M^{m-m_{0}}}\\ & \times & {\left(S^{max}-S\right)}^{n_{0}}\left(1-\frac{1}{M}\right)\sum_{n\geq n_{0}}\frac{{\left(S^{max}-S\right)}^{n-n_{0}}}{M^{n-n_{0}}}. \end{array}$$

If both series converge for any $S^{min}\leq S\leq S^{max}$, which is the case if and only if the condition $Z(=S^{max}-S^{min})<M$ is met, then we directly obtain

(17) $$\begin{array}{c} X\left(S;m_{0},n_{0}\right)={\left(1-\frac{1}{M}\right)}^{2}\frac{{\left(S^{max}-S\right)}^{m_{0}}}{1-\frac{S^{max}-S}{M}}\;\frac{{\left(S-S^{min}\right)}^{n_{0}}}{1-\frac{S-S^{min}}{M}}. \end{array}$$

In other words, the *M* parameter can always be chosen, so that the sums of both series in Equation (21) diverge for all *S* values. Thus, $m_{0},n_{0}\geq 1$ is the natural lower limit of $m_{0},n_{0}$, satisfying the condition of $X(S;m_{0},n_{0})$ disappearing for $S=S^{min},S^{max}$. We still assume more strongly that $m_{0},n_{0}\geq 2$, which has already been explained above.

For extensive systems, Equation (17) can be presented in a form that clearly shows the dependence of the *X* complexity on the number of entities *N*, simply replacing *S* entropy by Ns, where *s* is already *N*-independent specific entropy. Subsequently,

(18) $$\begin{array}{c} X\left(Ns;m_{0},n_{0}\right)={\left(1-\frac{1}{M}\right)}^{2}N^{m_{0}+n_{0}}\frac{{\left(s^{max}-s\right)}^{m_{0}}}{1-\frac{N}{M}\left(s^{max}-s\right)}\;\frac{{\left(s-s^{min}\right)}^{n_{0}}}{1-\frac{N}{M}\left(s-s^{min}\right)}. \end{array}$$

We emphasize that *X* does not scale with *N*, as opposed to partial measures of complexity.

**Figure 6:**
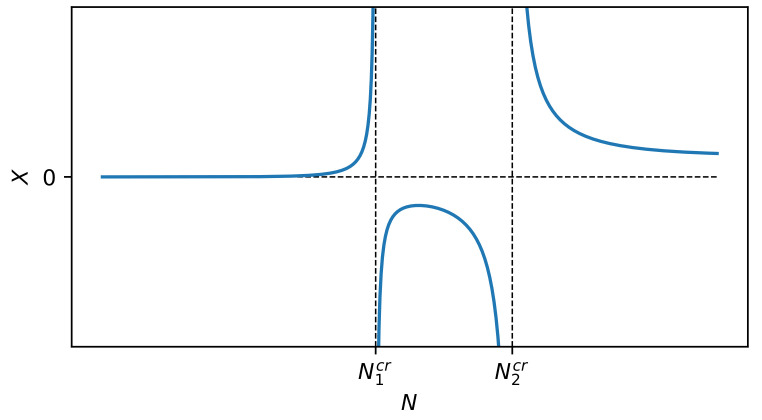
Dependence of the universal full measure of complexity *X* vs. number of entities *N* given by Equation (23). It should be emphasized that the full measure of complexity and its susceptibility have singularities in the same points. As one can see, we are dealing here with complexity barriers separating the phases/states of the system and the small and large number of objects forming them. The parameters we adopted here are as follows: M=30, $s^{min}=0,\;s^{max}=2,\;s=0.8,\;m_{0}=n_{0}=2$, hence, point $N_{cr}^{max}\left(s=0.8\right)=25$ and point $N_{cr}^{min}\left(s=0.8\right)=37.5$.

**Figure 7:**
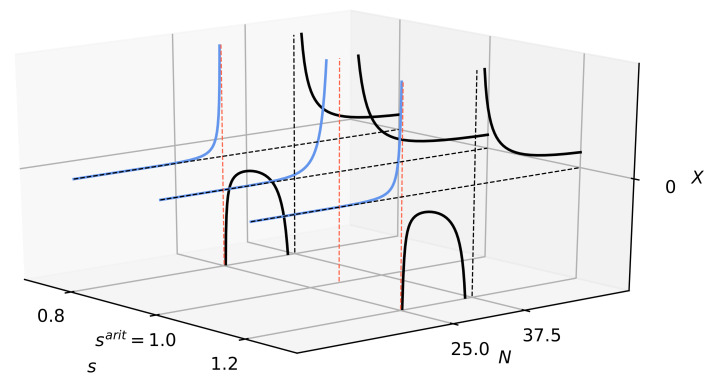
Dependence of the universal full measure of complexity *X* vs. number of entities *N* and specific entropy *s* given by Equation (23), for $m_{0},n_{0}\geq 1$. Notably, the full measure of complexity and its susceptibility have singularities at the same points $N_{cr}^{max}\left(s\right)$ and $N_{cr}^{min}\left(s\right)$. We are dealing here with complexity barriers separating the phases/states of the system and the small and large number of entities that form them. The parameters we adopted here are, as follows: M=30, $s^{min}=0,\;s^{max}=2,\;s=0.8,\;m_{0}=n_{0}=2$. These are the same parameters that we used to construct the plain plot in Figure 6.

In Figure 6 and Figure 7, we show the dependence of *X* on *N* (on the plane) and on *N* and *s* (in three dimensions), respectively. We obtained the singularities of full complexity, $N_{j}^{cr}\left(s\right),\;j=1,2$, as a result of the zeroing of denominators in the Equation (17) at nonzero numerators.

Note that, for M≫Z, both measures of complexity have approximate values X(S;m0,n0)≈CX(S;m0,n0). Important differences between these two measures only appear for Z/M close to 1, because only then does the denominator in Equation (17) play an important role. Of course, *M* is a free parameter, and possibly its specific value could be obtained from some additional (e.g., external) constraint.

In Figure 4, we compare the behaviour of the partial (black curve) and full (orange curve) measures of complexity, where we used the entropy instead of the specific entropy. Whether CX lies below or above *X* depends both on *M* parameter (determining the weight at which individual measures of partial complexity enter the full measure of complexity), and on the Z/M ratio.

We continue to determine the full entropic susceptibility of the full measure of complexity,

(19) $$\begin{array}{ccc} \chi_{X}\left(S;m_{0},n_{0}\right) & = & \frac{dX(S;m_{0},n_{0})}{dS}=\left(m_{0}+n_{0}\right)\left(S_{CX}^{max}-S\right)X\left(S;m_{0}-1,n_{0}-1\right)\\ & + & \frac{2}{M^{2}}X\left(S;m_{0},n_{0}\right)\frac{S-S^{arit}}{\left(1-\frac{S^{max}-S}{M}\right)\left(1-\frac{S-S^{min}}{M}\right)},\;m_{0},n_{0}\geq 1, \end{array}$$

where $S_{CX}^{max}$ is given here by Equation (4) but for $m=m_{0}$ and $n=n_{0}$. Notably, for the symmetric cases m=n and/or $m_{0}=n_{0}$, we have $S_{CX}^{max}=S_{X}^{max}=S^{arit}$, which are independent of $m,m_{0}$.

Similarly to the partial entropic susceptibility of a partial measure of complexity, we obtain the full entropic susceptibility of a full measure of complexity,

(20) $$\begin{array}{ccc} \chi_{X}\left(Ns;m_{0},n_{0}\right) & = & \frac{dX(S;m_{0},n_{0})}{dS}=\left(m_{0}+n_{0}\right)\;N\;\left(s_{CX}^{max}-s\right)X\left(Ns;m_{0}-1,n_{0}-1\right)\\ & + & \frac{2}{M^{2}}X\left(Ns;m_{0},n_{0}\right)N\frac{s-s^{arit}}{\left(1-\frac{N}{M}\left(s^{max}-s\right)\right)\left(1-\frac{N}{M}\left(s-s^{min}\right)\right)},\;m_{0},n_{0}\geq 1, \end{array}$$

where $s_{CX}^{max}=S_{CX}^{max}/N$, $s^{arit}=S^{arit}/N$, $s^{min}=S^{min}/N$, and $s^{max}=S^{max}/N$. The progression of susceptibility $\chi_{X}\left(S;m_{0},n_{0}\right)$, depending on *S*, for selected parameter values is shown in Figure 5. This progression course is similar to the analogous one that is presented in Figure 3.

Thus, the evolution of *X* is governed by an equation that is analogous to Equation (6), except that $\chi_{CX}$ present in that equation should be replaced by $\chi_{X}$ given by Equation (19). Therefore, we have

(21) $$\begin{array}{c} \frac{dX(S\left(t\right),m_{0},n_{0})}{dt}=\chi_{X}\left(S\left(t\right);m_{0},n_{0}\right)\frac{dS(t)}{dt}. \end{array}$$

The relationship between measures of complexity and time is implicit in our work—complexity indirectly depends on time through the dependence of entropy on time. It should be emphasized that the dependence of entropy on time is external in our approach—it can be taken into account based on additional modelling that is dedicated to specific real situations. We have already signalled this when discussing Equation (6).

### 2.7. Criticality in Extensive Systems

By using Equation (17), we show when the universal full measure of complexity diverges and, thus, the system enters a critical state. We assume that we are dealing with an extensive system, i.e., that Equation (17) can be represented as

(22) $$\begin{array}{c} X\left(Ns;m_{0},n_{0}\right)={\left(1-\frac{1}{M}\right)}^{2}N^{m_{0}+n_{0}}\frac{{\left(s^{max}-s\right)}^{m_{0}}}{1-\frac{N}{M}\left(s^{max}-s\right)}\times \frac{{\left(s-s^{min}\right)}^{n_{0}}}{1-\frac{N}{M}\left(s-s^{min}\right)},\;\frac{Nz}{M}<1, \end{array}$$

where entropy densities $s\left(=S/N\right),s^{min}\left(=S^{min}/N\right),s^{max}\left(=S^{max}/N\right)$ are (at most) slowly varying functions of the number *N* of elements making up the system and special entropy span $z=s^{max}-s^{min}$. As one can see, the measure *X* is divergent in two critical points $N_{cr}^{max}\left(s\right)=\frac{M}{s^{max}-s}$ and $N_{cr}^{min}\left(s\right)=\frac{M}{s^{min}-s}$, where $s^{min}<s<s^{max}$. Moreover, the susceptibilities given by Equations (19) and (20) diverge at the same points where measures of complexity given by Equations (17) and (18) diverge, which underlines the self-consistency of our approach.

Equation (22) can now be written in a form that explicitly includes both critical points (both physical and non-physical):

(23) $$\begin{array}{c} X\left(Ns;m_{0},n_{0}\right)={\left(1-\frac{1}{M}\right)}^{2}N^{m_{0}+n_{0}}\frac{{\left(s^{max}-s\right)}^{m_{0}}}{{\left(1-\frac{N}{N_{cr}^{max}\left(s\right)}\right)}^{\beta^{max}}}\times \frac{{\left(s-s^{min}\right)}^{n_{0}}}{{\left(1-\frac{N}{N_{cr}^{min}\left(s\right)}\right)}^{\beta^{min}}}, \end{array}$$

where critical exponents assume the mean-field values $\beta^{max}=\beta^{min}=1$. In this case, we could speak of two-criticality were it not for the fact that one of these criticalities is unphysical.

Figure 6 shows dependence $X(Ns;m_{0},n_{0})$ vs. *N* at fixed s=0.8. The values of parameters are shown there, while the specific entropy *s* is chosen so that the condition $s-s^{min}<s^{max}-s$ is satisfied (this is equivalent to a condition $s<s^{arit}$). This means that *s* is closer to $s^{min}$ than $s^{max}$. The existence of these divergences is a signature of criticality. However, the situation for borderline cases $s=s^{min}$ or $s=s^{max}$ changes rapidly—it is a different consideration.

Critical numbers of entities in the system $N_{cr}^{max}\left(s\right)$ and $N_{cr}^{min}\left(s\right)$ are determined by the ratio of the *M* parameter characterizing the hierarchy/cascade of scales in the system and the distance between entropy density *s* and its extreme values $s^{min}$ and $s^{max}$. The construction of these critical numbers resembles the canonical critical temperature structure for the Ising model in the mean-field approximation, where $\beta_{c}Jz=1$ (here $\beta_{c}=1/k_{B}T_{c}$ and $k_{B}$ is the Boltzmann constant). In our case, the role of the inverse temperature $\beta_{c}$ is played by $N_{cr}^{max}$ and $N_{cr}^{min}$, the role of the coupling constant *J* is 1/M, while the role of the mean coordination number *z* is played by $s^{max}-s$ and $s-s^{min}$, respectively.

The hierarchy is the source of criticality here. Criticality is an immanent feature of our full description of complexity. Nevertheless, in this work, we do not specify the sources of this hierarchy—it could be self-organized criticality or due to some other sources.

For the sake of completeness, note that the dependence on *N* of the partial measure of complexity is given by Equation (2). This means that for extensive systems this measure increases powerfully depending on N. Therefore, only the weighted infinite sum of these measures generates the existence of singularity.

Let us now consider in more detail the behaviour of $X(Ns;m_{0},n_{0})$ depending on *N* and *s*. A three-dimensional plot of Figure 7 will be helpful here. One can see how the mutual location of the singularities of $N_{cr}^{max}\left(s\right)$ and $N_{cr}^{min}\left(s\right)$ changes with the increase of s. From the situation of $s<s^{arit}$, in which $N_{cr}^{max}\left(s\right)>N_{cr}^{min}\left(s\right)$, through the situation when $s=s^{arit}$ in which $N_{cr}^{max}\left(s\right)=N_{cr}^{min}\left(s\right)$, up to the situation in which $N_{cr}^{max}\left(s\right)>N_{cr}^{min}\left(s\right)$ for $s>s^{arit}$.

It must be clearly stated that the area physically accessible is the one in front of the first singularity, which is further emphasized in Figure 7 by blue curves. Let us emphasize that the *N* range in which criticality occurs is sufficient to cover the corresponding values of *N* discussed in the literature to date, especially the Dunbar numbers [46,47,48,49] (e.g., N=5,15,50, and N=150). However, it should be noted that our view of complexity is complementary to that presented in the literature.

## 3. Finger Print of Complexity in Simplicity

Let us consider a perfect gas at a fixed temperature, which is initially closed in the left half of an isolated container. The partition is next removed, and the gas undergoes a spontaneous expansion. Here we are dealing (practically speaking) with an irreversible process, even for a small number of particles (at least the order of $10^{2}$).

Let us recall the definition of ’perfect gas’. It is a gas of particles that cannot ‘see’ each other, i.e., there are no interactions between them. Thus, from a physical point of view, it is a dilute gas at high temperature. We further assume that all of the particles have the same kinetic energy. A legitimate question is whether such a gas will expand after the partition is removed. We notice that the thermodynamic force is at work here, being roughly proportional to the difference in the number of particles in the right and left parts of the container. This force causes the expansion process. Thus, we are dealing with the simplest paradigmatic irreversible process [50]. The particles remain stuck in the final state and will not leave it (with accuracy subject to slight fluctuations in the number of particles in the right half of the container). Such a final state of the whole system is referred to as the equilibrium state. The simple coarse-grain description of the system allows us to introduce here the concept of configuration entropy.

Note that the macroscopic state of the system (generally, the non-equilibrium and non-stationary/relaxing one) can be described by the instantaneous number of particles in the left ($N_{L}\left(t\right)$) and right ($N_{R}\left(t\right)$) parts of the container, with $N=N_{L}\left(t\right)+N_{R}\left(t\right)$, where *N* is the fixed total number of particles in the container (isolated system). It allows for one to define the weight of the macroscopic state $\Gamma (N_{L}\left(t\right))$, also called thermodynamic probability. This is the number of ways to arrange the $N_{L}\left(t\right)$ particles in the left part of the container and $N_{R}\left(t\right)=N-N_{L}\left(t\right)$ in the right. Hence,

(24) $$\begin{array}{c} \Gamma \left(N_{L}\left(t\right)\right)=\frac{N!}{N_{L}\left(t\right)!\left(N-N_{L}\left(t\right)\right)!}. \end{array}$$

Here we do not distinguish permutations of particles inside each part of the container separately. We only take into account permutations of particles located in different halves of the container. This is because our resolution here is too small to observe the location of particles inside each container separately. Such a coarse-graining creates an information barrier: more information can mask the complexity of the system. We will not be able to see the complexity, because we will not be able to construct entropy. This creates a paradoxical situation: the surplus of information makes the task difficult and does not facilitate obtaining the insight into the system. Here we have an analogy with chaotic dynamics, where chaos is only visible in the Poincaré surface cross-section of the phase space and not in the entire phase space.

The configuration entropy at a given time *t* we define, as follows,

(25) $$\begin{array}{c} S\left(N_{L}\left(t\right)\right)=\ln\Gamma \left(N_{L}\left(t\right)\right), \end{array}$$

where $\Gamma (N_{L}\left(t\right))$ is given by Equation (24). The above expression can be used both for the equilibrium and non-equilibrium states.

It can be demonstrated using the Stirling formula that for large *N*, entropy *S* is reduced to the BGS form,

(26) $$\begin{array}{c} \ln\Gamma \left(N_{L}\left(t\right)\right)=-N\left[p_{L}\left(t\right)\ln p_{L}\left(t\right)+p_{R}\left(t\right)\ln p_{R}\left(t\right)\right]=Ns\left(t\right), \end{array}$$

where $p_{J}\left(t\right)\overset{\mathrm{def}.}{=}\frac{N_{J}\left(t\right)}{N},\;J=L,R$, and s(t) is a specific entropy. The law of entropy increase Equation (A8) is also fulfilled here, as expected.

We now prepare the equation for determining $N_{L}^{CX^{max}}$, i.e., the number of particles in the left part of the container that maximizes the partial complexity measure CX. To this end, we assume, for instance, the symmetric partial measure of complexity of the order of (m=2,n=2). Next, we substitute $N_{L}=N_{L}^{CX^{max}}$ into the both sides of Equation (25) and according to constitutive Equation (5), we equate Equation (25) to $S_{CX}^{max}$. Hence, we obtain a constitutive equation for the relaxing perfect gas,

(27) $$\begin{array}{c} S\left(N_{L}\left(t\right)=N_{L}^{{CX}^{max}}\right)=S_{CX}^{max}, \end{array}$$

where $N_{L}^{{CX}^{max}}$ is our sought quantity.

Now, we need to independently determine $S_{CX}^{max}$. Recall that the number of $N_{L}$ particles that maximize entropy is the number of $N_{L}^{eq}$ particles in the statistical/thermodynamic equilibrium state of the system. This number is equal to half of all particles in the container, i.e., $N_{L}^{eq}=N/2$. It can still be assumed (without reducing the general considerations) that $S^{min}=0$. Therefore,

(28) $$\begin{array}{c} S^{max}=S\left(N/2\right). \end{array}$$

However, from Equation (A5), we know that $S_{CX}^{max}=S^{max}/2$. By using it, we transform Equation (27) into the form,

(29) $$\begin{array}{c} S\left(N_{L}^{{CX}^{max}}\right)=\frac{1}{2}S\left(N/2\right). \end{array}$$

Equation (27) is an example of the general constitutive Equation (5), where $N_{L}^{CX^{max}}$ plays the role of $Y^{CX^{max}}$. This equation has the following explicit form,

(30) $$\begin{array}{c} {\left[\Pi_{j=1}^{N-N_{L}^{CX^{max}}}\left(1+\frac{N_{L}^{CX^{max}}}{j}\right)\right]}^{2}=\Pi_{j=1}^{N/2}\left(1+\frac{N}{2j}\right),\;\mathrm{for}\;n=m=2. \end{array}$$

Just deriving Equation (30) (see Appendix C for details) is the primary purpose of this example. This is a transcendental equation of which the exact analytical solution is unknown. When deriving Equation (30), we used the initial condition for the entropy that is, $S\left(t=0\right)=S^{min}=\ln\Gamma \left(N_{L}=N\right)=0$, which follows from Equations (24) and (25). Even for such a simple toy model, determining the partial measure of complexity is a non-trivial task, also because $N_{L}$ is different from N/2 (as we show below).

**Figure 8:**
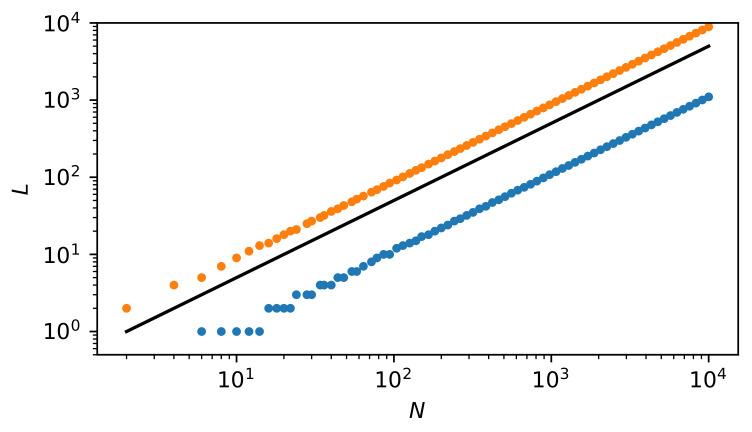
Dependence of $L(=N_{L}^{CX^{max}})$ vs. *N*. There are two solutions of Equation (30): one marked with blue circles and the other with orange ones. Above $N\approx 10^{2}$, both dependencies are linear, which is particularly clearly confirmed in Figure 9. That is, in a log-log scale, their slopes equal 1. However, in linear scale, the directional coefficients of these straight lines equal 0.11 and 0.89, respectively. This is clearly shown in Figure 9. Only the solution with orange circles is realistic, because the chance that 89% of particles will pass in a finite time to the second part of the container (as indicated by the solution marked with blue circles) is negligibly small. The black solid tangent straight line indicates a reference case $N_{L}^{CX^{max}}=N/2$.

The numerical solutions of Equation (30), i.e., the relationship of $N_{L}^{CX^{max}}$ to *N*, are shown in Figure 8 (for simplicity, *L* defining the vertical axis on the plot means $N_{L}^{CX^{max}}$). Both of the solutions (small circles above and below the solid straight line) show that $N_{L}^{CX^{max}}$ is significantly different from N/2. Thus, the most complex state is significantly different from the equilibrium state.

Having the $N_{L}^{{CX}^{max}}$ dependence on *N*, we can obtain the dependence of the partial measure of specific complexity $cx^{max}\overset{\mathrm{def}.}{=}CX^{max}/N^{m+n}$ on *N* order (m=2,n=2). We can write

(31) $$\begin{array}{c} cx^{max}={\left(\frac{s(N/2)}{2}\right)}^{4}={\left[\frac{1}{2N}\ln\left(\Pi_{j=1}^{N/2}\left(1+\frac{N}{2j}\right)\right)\right]}^{4}, \end{array}$$

as in our case $s^{max}=s\left(N/2\right)$ equals the logarithm of the right-hand side of Equation (30) divided by *N*. Notably, Equation (31) is based on Equation (A12).

**Figure 10:**
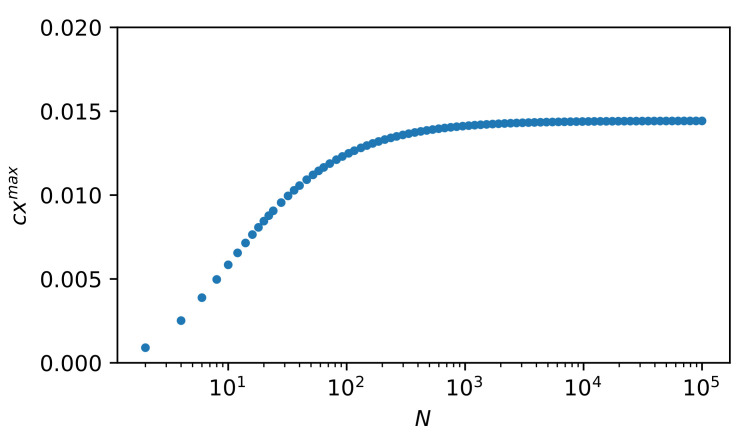
Dependence of $cx^{max}$ on *N* given by Equation (31). As one can see, $cx^{max}$ is a non-extensive function—it reaches the plateau for N≫1. For $N\approx 10^{4}$ the plateau is achieved with a good approximation. This is an important issue for researching complexity. Namely, systems can attain complexity already on a mesoscopic scale. It can be said that the curve’s inflection point (located near N=10) marks the beginning of the complexity stabilization region.

In Figure 10, we present the dependence of $cx^{max}$ on *N*. Quantity $cx^{max}$ is a non-extensive function—it reaches the plateau for N≫1. For $N\approx 10^{4}$ the plateau is achieved with a good approximation. This is important for researching complexity. Namely, systems can attain complexity already on a mesoscopic scale. Although the absolute value of the complexity measure is relatively small, it is evident and possesses a structure that is related to the current inflection point there (near N=10).

This example shows that even such a simple arrangement of non-interacting objects may have non-equilibrium non-stationary complexity. A necessary (but not sufficient) condition is the possibility of constructing entropy and the presence of a time arrow.

## 4. Concluding Remarks

In many recent publications [5,8,9,51] it is argued that entropy can be a direct measure of complexity. Namely, a smaller value of entropy indicates more regularity or lower system complexity, while its larger value indicates more disorder, randomness and higher system complexity. However, according to Gell–Mann, more disorder means less, and not more, system complexity. These two viewpoints are contradictory—this is a serious problem, which we have addressed.

Our motivation in solving the above problem was based on Gell–Mann’s view of complexity. This is because we fail to agree that the loss of information by the system as it approaches equilibrium increases its complexity; notably, $\Delta I(p^{eq},p^{eq})$ (see Appendix B for detail) takes its minimum value then, and complexity must decrease.

In addition, the differences between entropies in Equation (1) eliminate the useless dependence of complexity on the additive constant that may appear in the definition of entropy. It can be said that the system state with the highest complexity is the state most distant from all of the states of the system of lesser or no complexity.

Thus, in the sense of Gell–Mann, the measure of complexity should supply complementary information to the entropy or its monotonic mapping.

Therefore, in this work, we have presented a methodology which allows building a universal measure of complexity as a function of a system state based on non-linearly transformed entropy. This is a non-extensive measure. This measure should meet a number of conditions/axioms, which we have indicated in this work. A parsimonious example, of the simplest system with a small and a large number of degrees of freedom, is presented in order to support our methodology. As a result of this approach, we have shown that (generally speaking) the most complex are optimally mixed states consisting of pure states, i.e., of the most regular and most disordered, which the space of states of a given system allows. This also applies to the distinctive examples outlined in Appendix D and Appendix E (although this requires a redefinition of some variables and parameters).

We should pay attention to an essential issue regarding the definition of the phenomenological partial measure of complexity that is given by the Equation (1). This definition is open in the sense that if the description of complexity requires, for example, one additional quantity, then the Equation (1) takes on an extended form,

(32) $$\begin{array}{c} CX\left(S,E;m_{1},n_{1},m_{2},n_{2}\right)\overset{\mathrm{def}.}{=}{\left(S^{max}-S\right)}^{m_{1}}{\left(S-S^{min}\right)}^{n_{1}}{\left(E^{max}-E\right)}^{m_{2}}{\left(E-E^{min}\right)}^{n_{2}}\geq 0, \end{array}$$

whereby $E^{min}\leq E\leq E^{max}$ this new quantity is marked. This definition has still an open character. Specifically, this definition also allows (if the situation requires) the replacement of one quantity with another, e.g., entropy with free energy, or considering some derivatives (e.g., of the type $\frac{\partial S}{\partial E}$). Openness and substitutability should be the key features of the measure of complexity. Moreover, exponents $m_{j},\;n_{j},\;j=1,2,$ determine the order of complexity, i.e., its level or scale. We emphasize that the measure of complexity introduced can describe isolated and closed systems (although in contact with the reservoir), as well as open systems that can change their elements.

From Equations (13) and (32), we get the phenomenological universal full measure of complexity in the form, which extends Equation (17),

(33) $$\begin{array}{ccc} X(S,E;m_{1}^{0},n_{1}^{0},m_{2}^{0},n_{2}^{0}) & = & {\left(1-\frac{1}{M_{S}}\right)}^{2}\frac{{\left(S^{max}-S\right)}^{m_{1}^{0}}}{1-\frac{S^{max}-S}{M_{1}}}\frac{{\left(S^{min}-S\right)}^{n_{1}^{0}}}{1-\frac{S^{min}-S}{M_{1}}}\\ & \times & {\left(1-\frac{1}{M_{2}}\right)}^{2}\frac{{\left(E^{max}-E\right)}^{m_{2}^{0}}}{1-\frac{E^{max}-E}{M_{2}}}\frac{{\left(E^{min}-E\right)}^{n_{2}^{0}}}{1-\frac{E^{min}-E}{M_{2}}}\geq 0. \end{array}$$

The full measure of complexity is a weighted sum of partial measures of complexity across all complexity scales. As one can see, this full measure may contain singularities. They are the necessary signatures of criticality existing in the system. This meets the expectations presented in the literature.

Definitions of measures of complexity Equations (1) and (17) and their possible extensions are universal and useful. It is due to entropy that is associated not only with thermodynamics (Carnot, Clausius, Kelvin) and statistical physics (Boltzmann, Gibbs, Planck, Rényi, Tsallis), but also with the information approach (Shannon, Kolmogorov, Lapunov, Takens, Grassberger, Hantschel, Procaccia), and with the approach from the side of cellular automata (von Neumann, Ulam, Turing, Conway, Wolfram, et al.), i.e., with any representation of the real world using a binary string. Today, we already have several very effective methods for counting entropy of such strings, as well as other macroscopic characteristics sensitive to organization and self-organizing systems, as well as to their synchronization (synergy, coherence), competition, reproduction, adaptation—all of them sometimes having local and sometimes global characters.

Our definition of complexity also extends to meet research into the complexity of the biologically active matter. In this, especially research on the consciousness of the human brain can derive a fresh impulse. The point is that most researchers believe that the main feature of conscious action is a maximum complexity or even a critical complexity [52]. In our approach, it would be $CX^{max}$ and $X(N_{1}^{cr}s)$.

We hope that our approach will enable: (i) the universal classification of complexity, (ii) the analysis of a system critical behaviour and its applications, and (iii) the study of dynamic complexity. All of these constitute the background to the science of complexity.

## Figures and Tables

**Figure 9:**
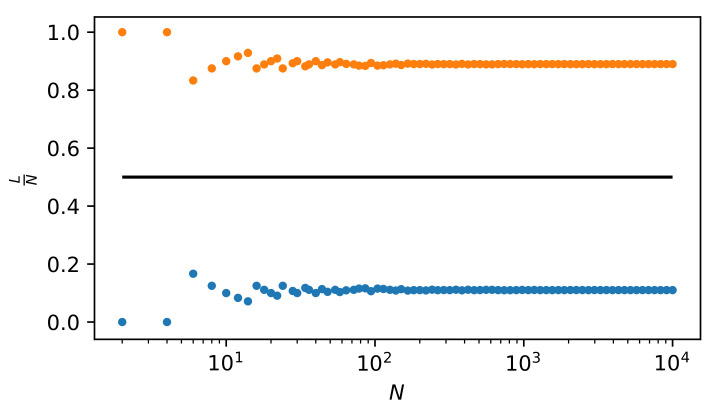
Directional coefficient of linear dependencies *L* vs. *N* as a function of *N*. For *N* greater than $10^{2}$, no *N*-dependence of this coefficient is observed. Both of the solutions (having L/N=0.11 and L/N=0.89) are mutually symmetric about the straight horizontal line L/N=1/2, but we only consider the solution L/N=0.89 to be realistic. The black horizontal straight solid line indicates a reference case $N_{L}^{CX^{max}}=N/2$.

**Figure A1:**
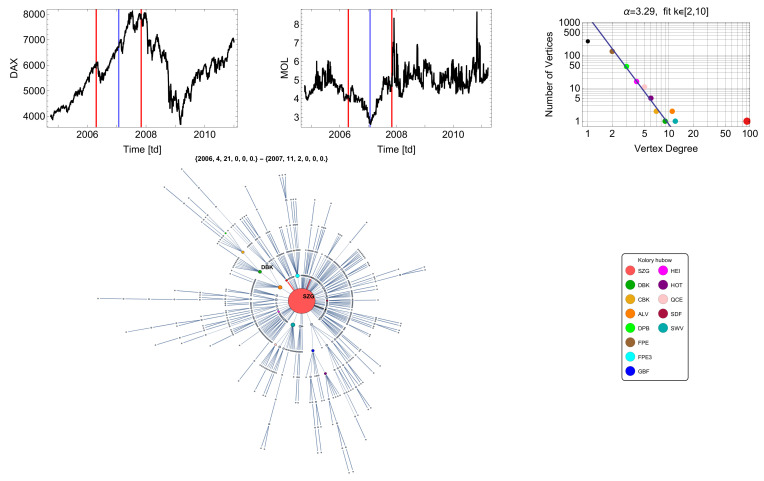
A snapshot picture of Monday 29 January 2007. The plot on the top left shows the DAX index in the years 2005–2010. This index is the emanation of the entire stock exchange. Of course, a computer analyzes quotations of all companies on this stock exchange that are in the range mentioned above. Two vertical red straight lines mark the scanning window, and its centre is marked by a blue line. The current window location, i.e., the beginning and end, are given dates just below the DAX and MOL plots. The same scanning window applied to the MOL indicator. The middle plot at the top shows the course of the widely used mean-occupation layer (MOL) [32]. The upper plot on the right shows the distribution of degrees of the vertices of the network shown below. The red circle on this plot, which stands out significantly from the power-law distribution, concerns the centre of the star (the most prominent red circle). The coloured circles in the legend and the abbreviations of the names mean the companies presented in the graph. The figure was taken from our publication [32].

**Figure A2:**
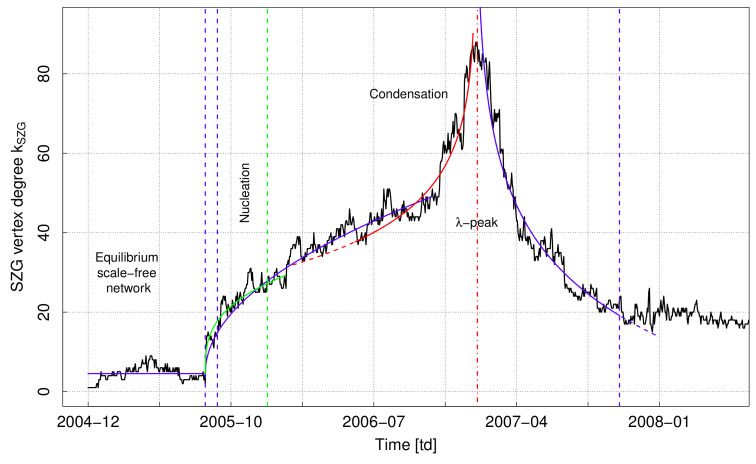
The temporal SZG vertex degree, $k_{SZG}$, vs. time. It forms the so-called λ-peak marked with a red vertical dashed straight line. It shows temporary edge condensation on the SZG node. The span of this peak is marked by the last blue vertical dashed straight lines. These lines are also plotted in Figure A3 below. The equilibrium scale-free networks are placed outside this area. The centre of this peak has been extrapolated on Thursday 25 January 2007—a deviation of two trading days from the result shown in Figure A1 is irrelevant here. The plot was taken from the publication [32] with the consent of the editors.

**Figure A3:**
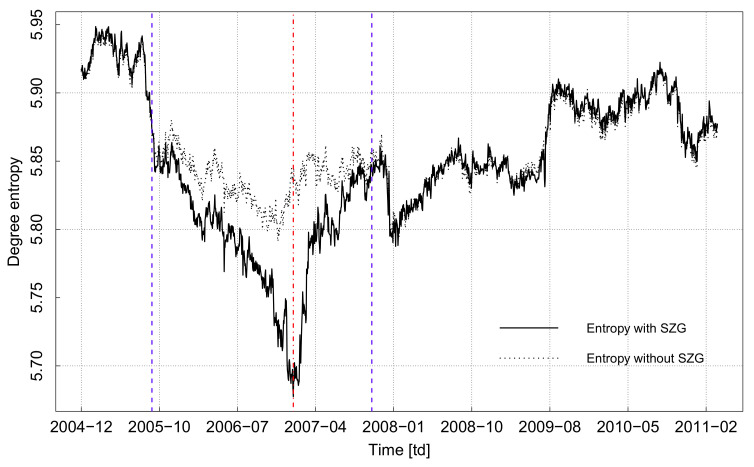
Degree entropy (defined in [32] by degree distributions) vs. time. The solid line marked entropy in the presence of the SZG vertex but dotted after removing this node. One can see the crucial role of this node in preparing the temporary network structure. The well-defined local absolute minimum of degree entropy is placed on Thursday, 25 January 2007. The plot was taken from the publication [32] with the consent of the editors.

**Figure A4:**
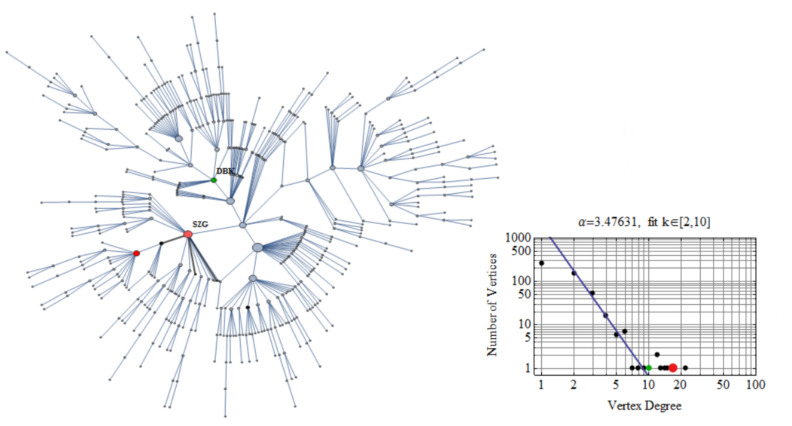
The snapshot picture of the Frankfurt Stock Exchange complex network on 21 December 2007. This network represents the most sophisticated state of the stock market, that is, $CX^{max}$ given by Equation (A31). One can see how much this network differs from that shown in Figure A1—e.g., the dominant central star has ceased to exist. The network structure is now skeletal.

## Appendix A. Properties of the Partial Measure of Complexity

It is worth paying attention to Equations (1)–(4). For a fixed span of *Z* and the order (m,n), there may still be systems of different complexities. The complexity description only using $CX^{max}$ is insufficient, because there can be many systems with the same span and order. However, we assume systems to be equivalent, i.e., belonging to the same complexity class (Z,m,n), if they have the same span at a given order. We can still distinguish them as, in general, they differ in the location of $S_{CX}^{max}$. We can say that a given class has a greater partial measure of complexity if it has a larger $CX^{max}$. In a given class, the system has a larger complexity if the system stays closer to $CX^{max}$, i.e., its current entropy *S* is closer to $S_{CX}^{max}$. For a given CX with Equation (1), we obtain (for each order (m,n)) two *S* solutions: one on the left and the other on the right of $CX^{max}$ (except when $S=S_{CX}^{max}$). Division into classes allows us to introduce an additional classification of complexity.

A distinction should be made between two cases of measuring complexity: (i) Z<m+n and (ii) Z>m+n. This is particularly evident when we consider the ratio of both types of complexity measures for m+n>1,

(A1) $$\begin{array}{c} \frac{CX^{max}\left(Z_{i}\right)}{CX^{max}\left(Z_{ii}\right)}={\left(\frac{Z_{i}}{Z_{ii}}\right)}^{m+n}<1, \end{array}$$

where $Z_{i}$ belongs to case (i), while $Z_{ii}$ to case (ii). Thus, the greater the exponent m+n, the greater the difference between $CX^{max}\left(Z_{ii}\right)$ and $CX^{max}\left(Z_{i}\right)$.

The alternate form of Equation (1),

(A2) $$\begin{array}{c} CX\left(\Delta \right)={\left(\frac{n}{n+m}Z+\Delta \right)}^{n}\;{\left(\frac{m}{n+m}Z-\Delta \right)}^{m}, \end{array}$$

where deviation $\Delta =\Delta \left(t\right)=S\left(t\right)-S_{CX}^{max}$ makes the operating of the CX coefficient easier in the vicinity of $S_{CX}^{max}$, where the parabolic expansion is valid. We then have:

(A3) $$\begin{array}{c} CX\left(\Delta \right)\approx CX^{max}\left[1-\frac{1}{2mn}{\left(\left(n+m\right)\frac{\Delta }{Z}\right)}^{2}\right]\approx CX^{max}\exp\left(-\frac{1}{2mn}{\left(\left(n+m\right)\frac{\Delta }{Z}\right)}^{2}\right), \end{array}$$

that is a Gaussian form, which has variance $\sigma^{2}=\frac{nm}{{\left(n+m\right)}^{2}}Z^{2}$. Only in the narrow range of *S* around $S_{CX}^{max}$ is the measure of complexity CX symmetrical regardless of the order (m,n).

In fact, only the location of the maximum of CX(S;m,n) is determined (for a given range of *S*) by the ratio of *m* to *n*. However, to have dependence of coefficient CX on entropy in the entire entropy range $S^{min}\leq S\leq S^{max}$, it is necessary to determine two extreme values of entropy ($S^{min}$ and $S^{max}$) and two exponents (*n* and *m*). In general, finding these parameters and exponents is still far from trivial because they have a contextual (and not a universal) character.

However, in a particular situation, when the maximum complexity is symmetrical, i.e., when m=n, we obtain

(A4) $$\begin{array}{c} S_{CX}^{max}=\bar{S}=\frac{S^{min}+S^{max}}{2} \end{array}$$

and

(A5) $$\begin{array}{c} CX^{max}={\left(\frac{Z}{2}\right)}^{2n}. \end{array}$$

Equation (1) of the partial measure of complexity applies both to single- and multi-particle problems because entropy can also be built even for a very long single-particle trajectory. Moreover, Equation (1) emphasizes our point of view that any evolving system for which one can introduce the concept of entropy and which has a state of thermodynamic equilibrium (for which entropy reaches a global maximum) contains at least a signature of complexity. For systems of negligible complexity, i.e., for which $S\approx S^{min}$ or $S\approx S^{max}$, the measure CX(S;m,n) is close to zero. This does not mean, however, that we cannot locate $S_{CX}^{max}$ near $S^{min}$ or $S^{max}$. It is then sufficient to have strongly asymmetric situations when n≪m or n≫m, respectively.

## Appendix B. Non-Stationary Entropies

In this Appendix, we sketch a non-stationary situation, which is what we are dealing with throughout this work. We are dealing with systems evolving to a state of statistical equilibrium, even from states far from statistical equilibrium.

Non-stationary entropy is understood to be entropy based on coarse-grained time-dependent probability distributions—this type of entropy is most often used [43,44,45,51]. A very characteristic example is the entropy class built on time-dependent probability distributions, $\left\{p_{j}\left(t\right)\right\}$, satisfying the master (Markovian) or M-equation, in the presence of detailed balance conditions. Thus, we are only considering systems evolving to statistical equilibrium.

Here we give two very characteristic (non-equivalent) examples of non-stationary entropies (more preciselly: one should talk about specific entropies). In addition, entropies given by Equations (A6) and (A7) belong to the category of relative entropies. Namely,

(A6) $$\begin{array}{c} S\left(t\right)=S_{0}\left[1-\sum_{j}p_{j}^{eq}f\left(\frac{p_{j}\left(t\right)}{p_{j}^{eq}}\right)\right] \end{array}$$

and

(A7) $$\begin{array}{c} S\left(t\right)=-S_{0}\ln\sum_{j}p_{j}^{eq}f\left(\frac{p_{j}\left(t\right)}{p_{j}^{eq}}\right), \end{array}$$

where $p_{j}\left(t\right)$ is a probability of finding a system in state *j* at time *t*, while $p_{j}^{eq}$ is a corresponding equilibrium probability. We are considering only discrete states here. The function $S_{0}f\left(x\right)\geq 0$, where domain 0≤x≤∞, is a non-negative convex function obeying $S_{0}\frac{d^{2}f}{dx^{2}}\geq 0$. It can be shown [45] that entropies defined in this way meet the law of entropy increase, i.e., its derivative

(A8) $$\begin{array}{c} \frac{dS(t)}{dt}\geq 0; \end{array}$$

therefore $S\left(t\right)\to S^{max}$ from below when $p_{j}\left(t\right)\to p_{j}^{eq}$, for any *j*. Equation (A8) is the key property of entropy. Let us add that at the limit $p_{j}\left(t\right)=p_{j}^{eq}$, for any *j*, entropy defined by Equations (A6) and (A7) disappears. In other words, these entropies are negative and grow to zero as the system tends to equilibrium.

It is worth paying attention to the possibility of defining generalized information gain, whereby this information gain is calculated here relative to the equilibrium distribution. We can write,

(A9) $$\begin{array}{c} \Delta I(p\left(t\right),p^{eq})=-S\left(t\right), \end{array}$$

where $p\left(t\right)=\{p_{j}\left(t\right)\}$ and $p^{eq}=\left\{p_{j}^{eq}\right\}$. Furthermore, entropy S(t) is closely related to partition function. Therefore, in this approach, the entropy is a base function.

Most often the function f(x) is selected in the form [45,56],

(A10) $$\begin{array}{c} f\left(x\right)=x^{\alpha },\;\alpha >1, \end{array}$$

coupled with a constant $S_{0}=\frac{1}{\alpha -1}$, where α can converge to 1. With these choices the entropy given by Equation (A6) is called Tsallis (relative) entropy and the entropy given by Equation (A7) Rényi (relative) entropy. Usually, the entropic index α is denoted by *q* in the case of Tsallis entropy.

Entropies given by Equations (A6) and (A7) converge, with the help of Equation (A10), to Kullback-Leibler entropy [9] when entropy index α→1. However, the Rényi and Tsallis entropies are essentially different for α≠1. The Rényi entropy is an additive function describing extensive systems, while the Tsallis is not. It is a non-additive function describing non-extensive systems.

Using relative entropy in the definition of complexity measures is productive. It is because other types of entropy can be derived from it, such as ordinary entropy (or Boltzmann-Gibbs-Shannon one) and conditional entropy.

## Appendix C. Derivation of the Constitutive Equation for Perfect Gas

The derivation of constitutive Equation (30) comes down to presenting both sides of Equation (29) in explicit form, shortening of common factors, proper organization and presentation. Accordingly, the left side of Equation (29) takes the form,

(A11) $$\begin{array}{ccc} S_{CX}^{max} & = & S\left(N_{L}^{CX^{max}}\right)=\ln\Gamma \left(N_{L}^{CX^{max}}\right)=\ln\frac{N!}{N_{L}^{CX^{max}}!\left(N-N_{L}^{CX^{max}}\right)!}\\ & = & \ln\frac{\left(N_{L}^{CX^{max}}+1\right)\left(N_{L}^{CX^{max}}+2\right)\cdot \ldots \cdot \left(N-1\right)N}{1\cdot 2\cdot 3\cdot \ldots \cdot \left(N-N_{L}^{CX^{max}}\right)}\\ & = & \ln\Pi_{j=1}^{N-N_{L}^{CX^{max}}}\left(1+\frac{N_{L}^{CX^{max}}}{j}\right), \end{array}$$

where the number of factors in the numerator and denominator is the same and equals $N-N_{L}^{CX^{max}}$.

As for the right side of Equation (29), we present it in an explicit form,

(A12) $$\begin{array}{ccc} \frac{1}{2}S\left(N/2\right) & = & \frac{1}{2}\ln\Gamma \left(N/2\right)=\frac{1}{2}\ln\frac{N!}{\left(\frac{N}{2}\right)!\left(\frac{N}{2}\right)!}\\ & = & \frac{1}{2}\ln\frac{\left(\frac{N}{2}+1\right)\left(\frac{N}{2}+2\right)\left(\frac{N}{2}+3\right)\cdot \ldots \cdot \left(\frac{N}{2}+\frac{N}{2}\right)}{1\cdot 2\cdot 3\cdot \ldots \cdot \frac{N}{2}}=\frac{1}{2}\ln\Pi_{j=1}^{N/2}\left(1+\frac{N}{2j}\right). \end{array}$$

Comparison of Equations (A11) and (A12) just leads to Equation (30).

## Appendix D. Entropies of Time Series—A Sketch

The entropy study of various time series is a crucial issue in system dynamics. The point is that the activity of the systems is perceived precisely through time series. The study of nonlinear time series is particularly important. Below we outline two essential methodologies for constructing entropy (including a multi-scale one). Then we show how to connect our complexity measure with these methodologies.

### Appendix D.1. Entropy of Embedded Time Series

Various time series from stock exchanges or Forex quotations are the central sources of empirical data available from financial markets. The main question is about the entropy of the time series and hence about the measure of time series complexity. Following Li et al. [51], we present a method of constructing entropy for a finite time series.

We consider the time series ${\left\{x_{j}\right\}}_{j=1}^{N}$ consisting of *N* elements $x_{j},\;j=1,2,\ldots$. From this we select 1≤i≤N−l+1 sub-series indexed by *i*. Each sub-series consists of *l* components defined as follows, $y_{i}^{l}\left(k\right):0\leq k\leq l-1$, where (for given indexes *i* and *l*) component $y_{i}^{l}\left(k\right)\overset{\mathrm{def}.}{=}x\left(i+k\right)$. As one can see, *m* here means the embedding dimension.

Moreover, two subsequent sub-series characterized by the same *l* have l−2 elements in common. In the collection of sub-series, which create *m*-dimensional vector space, one can enter a topology defined by the metric $d_{ij}$ defining the distance between arbitrary sub-series *i* and *j*. Then one can build a distribution (histogram), $p(d_{ij})$, of distances between vectors. Of course, the question of how to choose the embedding dimension *l* is fully justified. For example, one could assume that this dimension is equal to the correlation dimension [7]. However, we treat *l* as a free parameter, and we do not impose any additional restrictions on it. With the probability distribution, $p(d_{ij})$, one can build the entropy (for example, $S\left(l\right)=-\langle \ln p\left(d_{ij}\right)\rangle$) and hence the complexity of the time series (see Section 2.1 and Appendix B for details).

Similarly to the example with expanding gas in Section 3 and based on Equation (5), we can formulate the constitutive equation in the form

(A13) $$\begin{array}{c} S\left(l^{CX^{max}}\right)=\frac{\frac{1}{m}S\left(l^{max}\right)+\frac{1}{n}S\left(l^{min}\right)}{\frac{1}{m}+\frac{1}{n}}, \end{array}$$

where $S\left(l^{max}\right)=S^{max}$ and $S\left(l^{min}\right)=S^{min}$.

The transcendental Equation (A13) should be solved numerically due to the value of the unknown $l^{CX^{max}}$ sought. However, first, the values of $l^{min}$ and $l^{max}$ must be found (also numerically), which leads to the determination of $S^{min}$ and $S^{max}$, respectively. It allows us to determine $CX^{max}$. In the final step, one can (similar to that presented in Appendix B) find informative relationships of the above quantities $l^{CX^{max}}$, $l^{CX^{max}}/N$ and $CX^{max}$ from *N*.

This is all possible if one has sufficiently useful statistics, i.e., when $N-l+1\gg l\equiv \frac{N-1}{2}\gg l$.

### Appendix D.2. Multi-Scale Entropy

We can now proceed to define multi-scale complexity, but first we need to define multi-scale entropy or the hierarchy of entropies. For this purpose, we prepare the coarse-grained scheme. The primary time series consists of *N* elements. We divide it into n=Int[N/τ] non-overlapping intervals, where τ is the time horizon/scale, i.e., the number of time steps that we use to separate the elements of the first time series. Now we can build a new time series, of which the non-overlapping elements are defined as arithmetic means in subsequent intervals of τ,

(A14) $$\begin{array}{c} y_{j}^{\tau }=\frac{1}{\tau }\sum_{i=j\tau +1}^{(j+1)\tau }x_{i}\;,\;0\leq j\leq n-1. \end{array}$$

More can be said about the choice of τ using the (bilinear) autocorrelation function

(A15) $$\begin{array}{c} AC\left(t\right)\approx \frac{1}{n-t}\sum_{j=0}^{n-t}y_{j}^{\tau }y_{j+t}^{\tau }\;,\;n\gg t, \end{array}$$

if we are dealing with a stationary and a long time series. Then time $\tau =\tau_{c}$ can be considered, e.g., as the half-life of this function, i.e., $AC\left(\tau_{c}\right)\approx \frac{1}{2}AC\left(0\right)$. Other choices for τ can also be considered [43]. Note that the time series $\left\{y_{j}^{\tau }\right\}$ can consist of $\tau >\tau_{c}$ with statistically independent elements.

With time series dependent on the time scale τ, we can build scale-dependent entropy $S^{\tau }\left(t\right)$ and the corresponding complexity $CX(S^{\tau })$ by the corresponding methods presented in Appendix D.1. Thanks to this, for each scale τ separately (i.e., for each *n* separately), we can find quantities such as $n^{CX^{max}}$, $n^{CX^{max}}/N$ and $CX^{max}$.

### Appendix D.3. Elements of Deterministic Chaos: A Cyclically Kicked Damped Rotor

Deterministic chaos can be an example of the complexity of single-particle motion (i.e., the complexity of its phase space). It is caused by instability due to initial conditions. A typical example of this is a cyclically kicked damped rotor [20] or a damped pendulum with cyclic driving force [19]. Here we sketch this example.

The starting point is Newton’s equation for a rotor motion in the presence of viscous drag, which takes the form

(A16) $$\begin{array}{c} \frac{d^{2}\phi \left(t\right)}{dt^{2}}=-\gamma \;\frac{d\phi (t)}{dt}, \end{array}$$

where ϕ(t) is a time-dependent rotation angle of the rotor, γ is the viscous drag coefficient, and the moment of inertia is equal to the unit. The exact solution of Equation (A16) is based on the time-dependent exponential function.

Next, we enrich this equation with a non-linear impulse forcing force,

(A17) $$\begin{array}{c} F=\kappa f\left(\phi \right)\sum_{n=0}^{\infty }\delta \left(t-nT\right), \end{array}$$

where f(ϕ) is a non-linear function of ϕ and κ is its amplitude, while *T* is the period of this force. Hence, in the stroboscopic variables (or in the Poincaré representation) we obtain the Poincaré map,

(A18) $$\begin{array}{ccc} \omega_{n+1} & = & \exp\left(-\gamma T\right)[\omega_{n}+\kappa f\left(\phi_{n}\right)], \end{array}$$

(A19) $$\begin{array}{ccc} \phi_{n+1} & = & \phi_{n}+\frac{1-\exp(-\gamma T)}{\gamma }\left[\omega_{n}+\kappa f\left(\phi_{n}\right)\right], \end{array}$$

where $\omega =\frac{d\phi }{dt}$. The above set of recursive equations allows us to examine on the Poincaré surface both dissipative deterministic chaos (e.g., logistic or Henon mapping) and conservative (i.e., Chirikov mapping). This depends on the values of γ and κ parameters and the form of the function *f*.

Belief in the complexity of the phase space {ω,ϕ} of the system presented above is common. The complexity requires a knowledge of entropy. The total entropy of the system is the entropy of its long phase trajectory. However, we consider the complexity of the phase space structure mapped to the Poincaré surface, i.e., based on narrow entropy describing only this mapped structure. Obviously, this entropy depends on initial conditions and parameters assumed. Constructing entropy first requires defining a long time series. We have this time series as one consisting of *N* two-component elements ${\left\{\omega_{n},\phi_{n}\right\}}_{n=0}^{N-1}$. Indeed, the approach presented in Appendix D.1 can be used to calculate entropy.

The goal here is to have the entropy value $S_{CX}^{max}$ at which the complexity measure reaches the maximum value $CX^{max}$. It comes down to finding the dimension of the embedded subspace $l_{CX}^{max}$ for which the entropy $S=S_{CX}^{max}$. It is *l* that is the optimized parameter (in Section 3 it was the dynamic variable $N_{L}$). It can be expected that $l_{CX}^{max}$ is neither too small nor too large compared with the dimension *N* of the base space.

### Appendix D.4. Elements of Anomalous Diffusion

Let us consider as a typical example the deterministic dissipative motion of an extremely massive single molecule in a viscous fluid. This simple motion is described by the Newton’s dynamics, i.e., by the linear ordinary differential equation,

(A20) $$\begin{array}{c} \frac{du(t)}{dt}=-\gamma \;u\left(t\right), \end{array}$$

where u(t) is a time-dependent molecule velocity and γ is a constant friction coefficient. The exact solution of Equation (A20) is given by the time-dependent exponential function.

We are now moving from a deterministic level to a stochastic level by a relatively small extension of Equation (A20). That is, we extend Equation (A20) so as to obtain the retarded general Langevin equation (GLE) [50],

(A21) $$\begin{array}{c} \frac{du(t)}{dt}=-\int_{0}^{t}\gamma \left(t-t^{\prime }\right)u\left(t^{\prime }\right)dt^{\prime }+\frac{R(t)}{m}, \end{array}$$

where γ(t) is the retarded friction coefficient, R(t) is a random force of a thermal origin, i.e., caused by the random action of fluid particles, and *m* is the mass of the molecule. Notably, the retardation is present only when the mass of the molecule suspended in the fluid is not too heavy.

Equation (A21), which is the essence of the Ornstein-Uhlenbeck (OU) generalized theory of Brownian motion, takes into account both the feedback effect associated with the reverse fluid flow pushed by the molecule and the erratic nature of the molecule’s motion. Although it is still a linear equation relative to *u*, the velocity the autocorrelation function, C(t), is no longer expressed by a simple exponential, but exhibits a slower, power-law decay,

(A22) $$\begin{array}{c} C\left(t\right)\propto \frac{1}{t^{2-\alpha }},\;\alpha =\frac{1}{2}, \end{array}$$

for a long time, which is indeed more realistic. Equation (A22) is a central result of OU generalized theory. As one can see, a relatively small extension of Equation (A20)—small because it still leaves this equation in the domain of linear equations relative to *u*—led to decay according the power law. Such a law is an essential attribute of a complex system. This algebraic fat tail was noticed for the first time by Adler and Wainwright in molecular-dynamic simulation of hard spheres’ fluid [53], at least at intermediate fluid densities. Equation (A22) is the result of a cooperative phenomenon in the form of a positive feedback arising in the system between the molecule suspended in the fluid and the particles of the fluid. The non-linear nature of this coupling in time (that is the nonlinear dependence of γ on time) is contained in the integral kernel of Equation (A21). There is a wide class of physical problems that can be modelled using this equation [28,54] (for almost arbitrary α). Equation (A21) is the first level of complexity here.

Note that the direct consequence of Equation (A22) is the sub-diffusive behaviour of the suspended molecule, i.e., for long times the variance of the stochastic process *X* (*t*) (without a drift) takes the form,

(A23) $$\begin{array}{c} \langle X{\left(t\right)}^{2}\rangle \propto t^{\alpha }. \end{array}$$

Now one can ask a question about what the distribution family with the variance given by the above formula looks like. The answer is almost instant namely,

(A24) $$\begin{array}{c} P\left(X,t\right)=\frac{1}{t^{\alpha /2}}f\left(\frac{|X|}{t^{\alpha /2}}\right), \end{array}$$

which is a positive and normalized time-dependent probability distribution, where scaling function *f* is almost an arbitrary one.

Results Euqation (A23) and (A24) became the inspiration for extensive research on anomalous diffusion [28]. For example, by taking restrictions on the shape exponent α, i.e., α can be generally different from 1/2. This type of extension permits realistic considerations [28].

#### Characteristic Examples

Here we show how comprehensive the probability distribution given by Equation (A24) is.

**Example** **A1.**
*Brownian and Gaussian random walk.*

Let us assume for α=1,

(A25) $$\begin{array}{c} f\left(\frac{|X|}{t^{\alpha /2}}\right)=\frac{1}{\sqrt{2\pi }}\exp\left(-{\frac{X}{2t}}^{2}\right). \end{array}$$

As one can see, the variance of the distribution given by Equation (A24) with the substitution given by Equation (A25) satisfies Equation (A23)—it is a linear function of time, as it should be. This example is our reference case.

**Example** **A2.**
*Brownian and non-Gaussian random walk.*

Let us now assume that still α=1 but

(A26) $$\begin{array}{c} f\left(\frac{|X|}{t^{\alpha /2}}\right)=\frac{1}{2}\exp\left(-\frac{|X|}{t^{1/2}}\right), \end{array}$$

that is, probability distribution *P* is a Laplace distribution. Despite this, the variance of this distribution remains a linear function of time. Here we are dealing here with a Brownian and Laplace random walk.

**Example** **A3.**
*Non-Brownian and Gaussian random walk.*

Let us here assume that

(A27) $$\begin{array}{c} f\left(\frac{|X|}{t^{\alpha /2}}\right)=\frac{1}{\sqrt{2\pi }}\exp\left(-\frac{X^{2}}{2t^{\alpha }}\right), \end{array}$$

where α is an arbitrary shape exponent. As one can see, the variance is here (in general) a non-linear function of time: it can be both sub- and super-linear. Thus, we are dealing here with the fractional Brownian motion.

**Example** **A4.**
*Non-Brownian and Non-Gaussian random walk.*

Let us consider the Weibull distribution—this gives *f* function in the form,

(A28) $$\begin{array}{c} f\left(\frac{X}{t^{\alpha /2}}\right)=\kappa {\left(\frac{X}{t^{\alpha /2}}\right)}^{\kappa -1}\exp\left(-{\left(\frac{X}{t^{\alpha /2}}\right)}^{\kappa }\right),\;X\geq 0, \end{array}$$

where κ>1. The variance for Weibull distribution is given by Equation (A23). This means that we can model both sub-diffusion (when α<1) and super-diffusion (when α>1) using the Weibull distribution. Of course, with this distribution one can also model a Brownian (when α=1) but non-Gaussian random walk.

### Appendix D.5. Nonequilibrium Configuration Entropy

We are now constructing configuration entropy (i.e., based on configuration rather than phase space). We first discretize the space, i.e., the *X* axis. Let the size of the discretization step be ΔX. The time-dependent probability of finding a stochastic process at time *t* in the *n*th cell with size ΔX is,

(A29) $$\begin{array}{c} p_{n}\left(\Delta X,t\right)=\int_{n\Delta X}^{(n+1)\Delta X}P\left(X,t\right)dX, \end{array}$$

where free parameter ΔX here defines the level of coarse-graining. Indeed, we use this probability in Equations (A6) or (A7). The equilibrium probability needed there $p_{n}^{eq}\left(\Delta X\right)=p_{n}\left(\Delta X,t\to \infty \right)$ constructs in the usual way. That is, we confine the system to a very large but finite size. Then time *t* is going to infinity. Next, both probabilities (the nonequilibrium for finite time *t* and equilibrium probability) substitute to Equations (A6) or Equation (A7). Finally, the system goes to the thermodynamic limit.

The goal is to find ΔX size equal to $\Delta X_{CX}^{max}$ that maximizes CX, that is $CX^{max}$. This can only be done by numerical means.

We emphasize that Equation (A21) is the dynamic basis of the (stochastic) Fokker–Planck equation [45] as well as the Langevin [55] fractal equation and hence the Fokker-Planck [28] fractal equation. The same Equation (A21) is a springboard to move to higher levels of complexity.

## Appendix E. Dynamic Self-Organisation on a Complex Network—A Sketch

It is hard to imagine not using complex network technologies to analyze collective processes in the socio-economic world. One of the fascinating sources of complexity is its capacity for the self-organization—spontaneous (of endogenic) character or stimulated (of exogenic) character. We consider the dynamic phase transition here, in which the network of the stock market companies evolves towards a characteristic big-star structure—it condensates [6,32].

### Appendix E.1. Minimal Spanning Tree of the Frankfurt Stock Exchange

Here we consider the canonical Minimal Spanning Tree (MST) of the Frankfurt Stock Exchange (FSE) companies belonging to the widely-exploited class of correlation complex networks (the content of this section was created with the participation of Mateusz Wiliński). They describe well the dynamics of relationships between companies, that is the evolution of the MST. The MST is a very simple type of a complex network that, although it resets the clustering rate, provides a lot of important information about the structure and dynamics of real networks.

For example, in Figure A1, we present the self-organized structure of the Frankfurt Stock Exchange as of 29 January 2007. As one can see, the prominent star centred at SALZGITTER (SZG) AG-Stahl und Technologie company (the network node marked by the red circle located at the centre of the network) dominates the stock market structure. The middle plot on the top and the plot on the right there correctly reflect this type of behaviour. The former plot clearly shows the local minimum determining the size of the average coordination zone of the SZG node. Its average radius (i.e., the MOL equals about 2.5 separation steps) confirms that the SZG company is the centre of the star. The last plot shows a red circle with a multiplicity of 1 and a degree close to 100, which represents the SZG node. This node is an outlier—an excellent example of the appearance of a super-extreme event, i.e., dragon-king event [29,30,31].

The structure dynamics of the dragon-king is presented in Figure A2. That is, we presented there the dependence of the degree of this node on time, yielding a very meaningful λ peak characterizing here a structural condensate (for details see [32]).

In Figure A3 we compare empirical degree entropies $S\left(t\right)=-\sum_{k}P\left(k\right)\ln P\left(k\right)$, where P(k) defines the empirical degree distribution, versus time in the presence and absence of SZG company on the network. In Figure A1, the upper plot on the right shows an example of the (non-normalized) distribution, P(k), where *k* means the degree of a node. Various nodes are marked here with coloured circles defined in the legend. As one can see, the presence of the SZG node significantly changes the structure of the MST network. It is worth determining the complexity of such a network.

### Appendix E.2. Analysis for Case Z<1

We note that the extreme entropy values, for the data shown in Figure A3, are as follows: $S^{min}(t=$ 2007-01-25) = 5.690 and $S^{max}(t=$ 2005-03-15) = 5.948, which gives a span of Z=0.258<1. Substituting these numbers into Equations (A4) and (A5) we get the entropy of the order of (m=2,n=2),

(A30) $$\begin{array}{c} S_{CX}^{max}=5.819, \end{array}$$

and complexity measure of the same order

(A31) $$\begin{array}{c} CX^{max}=2.769\;10^{-4}. \end{array}$$

From the plot data: ‘Entropy with SZG ’in Figure A3, dates corresponding to the entropy given by Equation (A30) can be read. There are many of them—we chose three particular ones: 6 January 2006, 21 December 2007, 15 April 2008. All of them concern the most complex networks.

Figure A4 shows the network corresponding to the second date. One can see how much it differs from the least complex shown in Figure A1. This Figure shows a mixture of few mini stars with a degree not greater than about 20 (but no central one with degree almost 100, as shown in Figure A1). It is indicated by the degree distribution on the right-hand side of the plot, besides several skeletons and developed cascades arranged there in a somewhat disordered way. It is a richer and more disordered structure—more complex than that shown in Figure A1.

## References

[1] Nicolis G., Nicolis C. (2012). Foundations of Complex Systems. Emergence, Information and Prediction.

[2] Kwapień J., Drożdż S. (2012). Physical approach to complex systems. Phys. Rep..

[3] Dorogovtsev S.N., Goltsev A.V. (2008). Critical phenomena in complex networks. Rev. Mod. Phys..

[4] Albert R., Barabási A.-L. (2002). Statistical mechanics of complex networks. Rev. Mod. Phys..

[5] Pincus S.M. (1991). Approximate entropy as a measure of system complexity. Proc. Natl. Acad. Sci. USA.

[6] Dorogovtsev S.N. (2010). Lectures on Complex Networks.

[7] Grassberger P., Procaccia I. (1983). On the characterization of strange attractors. Phys. Rev. Lett..

[8] Richman J.S., Moorman J.R. (2000). Physiological time-series analysis using approximate entropy and sample entropy. Am. J. Physiol. Heart Circ. Physiol..

[9] Prehl J., Boldt F., Essex C.H., Hoffmann K.H. (2013). Time evolution of relative entropies for anomalous diffusion. Entropy.

[10] Thurner S., Hanel R., Klimek P. (2018). Introduction to the Theory of Complex Systems.

[11] Popiel N.J.M., Khajehabdollahi S., Abeyasinghe P.M., Riganello F., Nichols E., Owen A.M., Soddu A. (2020). The Emergence of Integrated Information, Complexity, and ‘Consciousness’ at Criticality. Entropy.

[12] https://en.wikipedia.org/wiki/Complexity.

[13] Thurner S., Hanel R., Gell-Mann M. (2014). How multiplicity of random processes determines entropy and the derivation of the maximum entropy principle for complex systems. Proc. Natl. Acad. Sci. USA.

[14] Grassbereger P. (1986). Toward a quantitative theory of self-generated complexity. Int. J. Theor. Phys..

[15] Bialek W., Nemenmana I., Tishby N. (2001). Complexity through nonextensivity. Phys. A.

[16] Prokopenko M., Boschetti F., Ryan A.J. (2008). An information-theoretic primer on complexity, self-organization, and emergence. Complexity.

[17] Borda M. (2011). Fundamentals in Information Theory and Coding.

[18] Crutchfield J.P. (2012). Between order and chaos. Nat. Phys..

[19] Baker L.G., Gollub J.P. (1996). Chaotic Dynamics: An Introduction.

[20] Schuster H.G. (1988). Deterministic Chaos. An Introduction.

[21] Henkel M., Hinrichsen H., Lübeck S. (2008). Non-Equilibrium Phase Transitions. Volume I: Absorbing Phase Transitions.

[22] Wolfram S. (2002). A New Kind of Science.

[23] Gell-Mann M. (2002). Plectics: The study of simplicity and complexity. Europhysicsnews.

[24] Gell-Mann M., Lloyd S. (1996). Information measures, effective complexity and total information. Complexity.

[25] Gell-Mann M., Lloyd S., Gell-Mann M., Tsallis C. (2004). Effective complexity. Nonextensive Entropy: Interdisciplinary Applications.

[26] Ay N., Müller M., Szkoła A. (2010). Effective complexity and its relation to logical depth. IEEE Trans. Inf. Theory.

[27] Gell-Mann M., Freeman W.H. (1994). The Quark and the Jaguar: Adventures in the Simple and the Complex.

[28] Kutner R., Masoliver J. (2017). The continuous time random walk still trendy: Fifty-year history, state of art, and outlook. Eur. Phys. J..

[29] Sornette D. (2009). Dragon-kings, black swans and the prediction of crises. Int. J. Terraspace Sci. Eng..

[30] Sornette D., Ouillon G. (2012). Dragon-kings: Mechanisms, statistical methods and empirical evidence. Eur. Phys. J. Spec. Top..

[31] Wiliński M., Sienkiewicz A., Gubiec T., Kutner R., Struzik Z.R. (2013). Structural and topological phase transition on the german stock exchange. Phys. A.

[32] Wiliński M., Szewczak B., Gubiec T., Kutner R., Struzik Z.R. (2015). Temporal condensation and dynamic *λ*-transition within the complex network: An application to real-life market evolution. Eur. Phys. J. B.

[33] Kozłowska M., Denys M., Wiliński M., Link G., Gubiec T., Werner T.R., Kutner R., Struzik Z.R. (2016). Dynamic bifurcations on financial markets. Chaos Solitons Fractals.

[34] Jakimowicz A. (2020). The role of entropy in the development of economics. Entropy.

[35] Jakimowicz A. (2016). Fundamental sources of economic complexity. Int. J. Nonlinear Sci. Numer..

[36] Rossler J.B. (2008). Econophysics and economic complexity. Adv. Complex Syst..

[37] Rossler J.B. (2016). Entropy and econophysics. Eur. Phys. J. Spec. Top..

[38] Zambelli S., George D.A.R. (2012). Nonlinearity, Complexity, and Randomness in Economics: Towards Algorithmic Foundations for Economic.

[39] Kutner R., Ausloos M., Grech D., Di Matteo T., Schinckus C.H., Stanley H.E. (2019). Econophysics and sociophysics: Their milestones & challenges. Physica A.

[40] Gell-Mann M. (1995). What is Complexity?. Complexity.

[41] Bertin E. (2012). A Concise Introduction to the Statistical Physics of Complex Systems.

[42] Jaynes E.T. (1965). Gibbs vs boltzmann entropies. Am. J. Phys..

[43] Beck C.H., Schlögl F. (1995). Thermodynamics of Chaotic Systems. An Introduction.

[44] Tsallis C. (1988). Possible generalization of boltzmann-gibbs statistics. J. Stat. Phys..

[45] Van Kampen G. (2007). Stochastic Processes in Physics and Chemistry.

[46] Bahcall S. (2019). Loonshots: How to Nurture the Crazy Ideas that Win Wars, Cure Diseases, and Transform Industries.

[47] West G. (2017). Scale: The Universal Laws of Life, Growth, and Death in Organisms, Cities, and Companies.

[48] Dunbar R.M. (1998). The social brain hypothesis. Evol. Antropol..

[49] Acharjee S., Bora B., Dunbar R.I.M. (2020). On M-polynomials of dunbar graphs in social networks. Symmetry.

[50] Kubo R., Toda M., Hashitsume N. (1985). Statistical Physics II. Nonequilibrium Statistical Mechanics.

[51] Li P., Liu C., Li K., Zheng D., Liu C., Hou Y. (2015). Assessing the complexity of short-term heartbeat interval series by distribution entropy. Med. Biol. Eng. Comput..

[52] Sornette D. (2000). Critical Phenomena in Natural Sciences. Chaos, Fractals, Selforganization and Disorder: Concepts and Tools.

[53] Alder B.J., Wainwright T.E. (1970). Decay of the velocity autocorrelation function. Phys. Rev. A.

[54] Bunde A., Havlin S. (1996). Fractals and Disordered Systems, Second Revised and Enlarged Edition.

[55] Camargo R.F., Chiacchio A.O., Charnet R., de Oliveira C.E. (2009). Solution of the fractional Langevin equation and the Mittag–Leffler functions. J. Math. Phys..

[56] Wehrl A. (1978). General properties of entropy. Rev. Mod. Phys..

